# Extracellular microparticles derived from hepatic progenitor cells deliver a death signal to hepatoma-initiating cells

**DOI:** 10.1186/s12951-022-01280-5

**Published:** 2022-02-14

**Authors:** Xiaojuan Hou, Wenting Liu, Xue Yang, Changchun Shao, Lu Gao, Li Zhang, Lixin Wei

**Affiliations:** 1 Tumor Immunology and Gene Therapy Center, Third Affiliated Hospital of Second Military Medical University, 225 Changhai Road, Shanghai, 200438 China; 2 Clinical Research Unit, Changhai Hospital, Naval Medical University, 168 Changhai Road, 200433 Shanghai, China

**Keywords:** Extracellular microparticles, Hepatic progenitor cells, Death signal, Hepatoma-initiating cells, Hepatocarcinogenesis

## Abstract

The malignant transformation of normal resident hepatic stem/progenitor cells has a critical role in hepatocarcinogenesis and the recurrence of hepatocellular carcinoma (HCC). We defined such hepatic progenitor cells as hepatoma-initiating cells. An efficient strategy is required to target and kill the hepatoma-initiating cells. We isolated extracellular microparticles (MPs) derived from apoptotic hepatic progenitor cells (HPCs) and tested their ability to inhibit hepatocarcinogenesis. Extracellular MPs were isolated from HPCs, hepatocytes and liver tumor cells. Their effects on tumor growth were investigated in rat primary HCC models, in which hepatocarcinogenesis is induced by diethylnitrosamine (DEN). The extracellular MPs derived from apoptotic HPCs, apoptotic hepatocytes and apoptotic liver tumor cells were similar in morphology, diameter and zeta potential. However, they had different antitumor effects. In DEN-exposed rats, only the MPs derived from apoptotic HPCs effectively inhibit hepatocarcinogenesis. In vitro and in vivo analyses confirmed that HPCs preferentially take up MPs derived from apoptotic HPCs compared to MPs from other liver cell types. Proteomic analysis of MPs from apoptotic HPCs showed enrichment of proteins involved in cell death pathways. Thus, HPC-derived MPs contain a death signal to induce the killing of hepatoma-initiating cells. Our findings provide evidence that a death signal encapsulated in HPC-derived extracellular microparticles can efficiently clear hepatoma-initiating cells and prevent hepatocarcinogenesis.

**Graphical Abstract:**

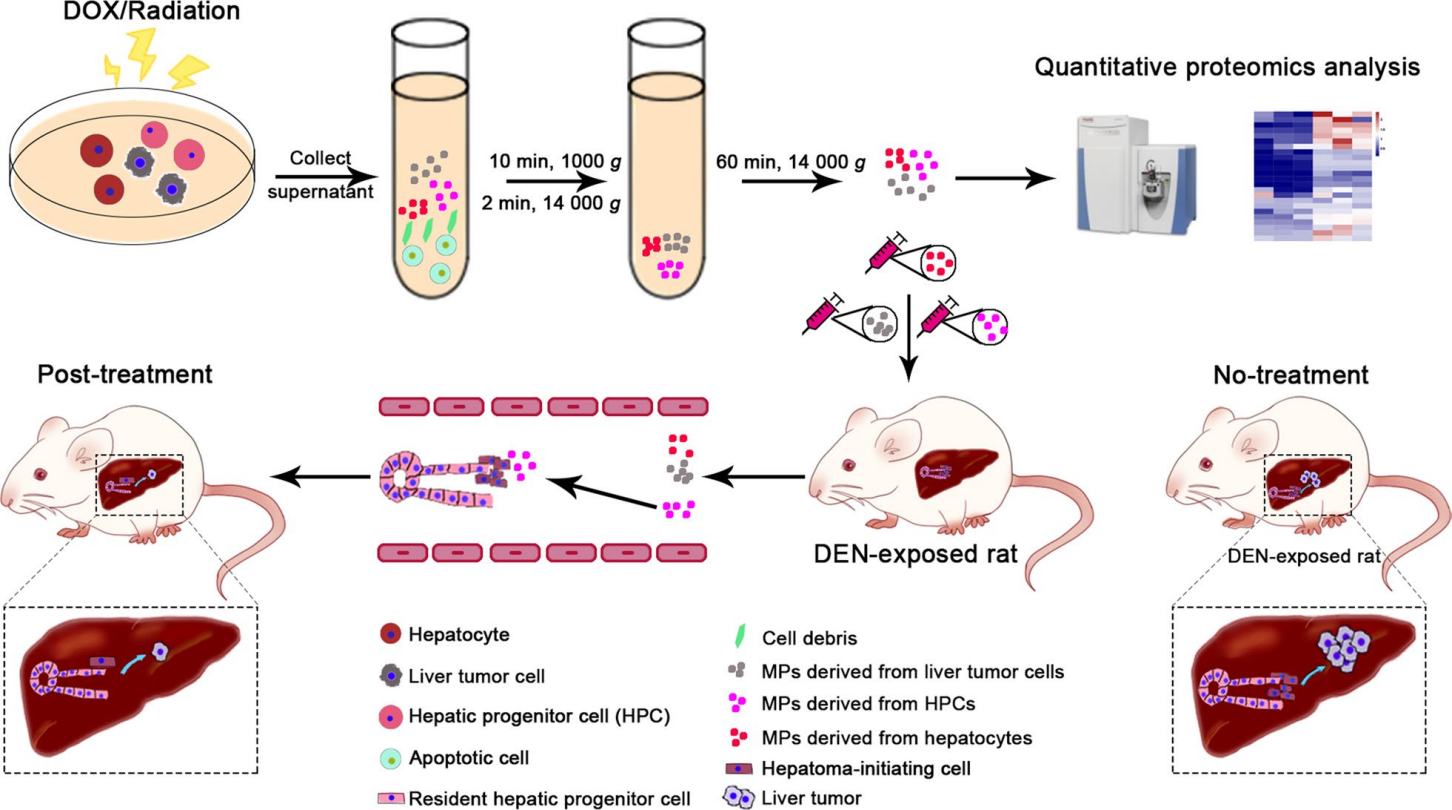

**Supplementary Information:**

The online version contains supplementary material available at 10.1186/s12951-022-01280-5.

## Introduction

Hepatocellular carcinoma (HCC), one of the most common malignant tumors, is an important health problem worldwide [1]. Liver cancer is estimated to be the fourth leading cause of cancer-associated deaths in the world [2], and its incidence is increasing [3]. Surgical resection only has therapeutic value in early-stage HCC patients [4, 5]. Treatment failure occurs frequently and is mainly caused by relapse and metastasis [4, 6]. Extensive proliferation and abnormal differentiation of hepatic progenitor cells (HPCs) are closely correlated with hepatocarcinogenesis [7, 8].

In our previous studies, we also found that the activation and malignant transformation of HPCs promote hepatocarcinogenesis and HCC recurrence [9–12]; thus, we defined such HPCs as hepatoma-initiating cells. Scavenging of these hepatoma-initiating cells is considered as an effective method for the prevention of hepatocarcinogenesis. In the field of cancer treatment, especially for liver cancer treatment, nanoparticles have been explored as drug delivery systems for several years. Nanoparticles inherently target the liver in a passive manner. Various nanoparticles, including lipid-based nanoparticles, metal-based nanoparticles, polymeric nanoparticles and dendrimers, have been used for targeted drug delivery in liver cancer treatment [13, 14]. However, these artificial nanoparticles may not be soluble in biological matrices, and they may possess potential toxicity. Thus, encapsulating the therapeutic reagent within a host-derived nanoparticle may minimize several side-effects. In recent years, extracellular microvesicles have attracted considerable attention as carriers of therapeutic reagents.

Either spontaneously or in response to stimuli, cells change their cytoskeleton and encapsulate cytosolic contents within cellular membranes to form microvesicles, which are then released into the surroundings [15, 16]. Such naturally formed extracellular microvesicles, which are 0.2–1 μm in size, are called microparticles (MPs). They selectively sort and transfer biological signals including signaling proteins, bioactive lipids or genetic material from donor cells to recipient cells. The same cell can release different MPs under different stimuli. For example, endothelial cells release qualitatively and quantitatively distinct MPs during activation compared to apoptosis [17]. Interactions of MPs with target cells can have various effects on the recipient cells depending on the bioactive signals in MPs. For instance, MPs isolated from patients with myocardial infarction were shown to induce endothelial dysfunction, while MPs derived from healthy subjects did not affect endothelial function [18, 19]. This suggests that the MP components and content specify biological function. Motivated by these lines of evidence, we reasoned that a “death signal” transferred by MPs may induce the death or apoptosis of recipient cells.

In nature, there exists the phenomenon of “like-dissolves-like”. Based on this concept, MPs derived from HPCs may be taken up preferentially by cells that originate from HPCs. Thus, to target hepatoma-initiating cells, MPs from HPCs can be considered as a carrier. It has also been shown that stem cells are capable of producing and shedding an abundance of MPs that may act as a carrier to deliver genetic and protein information [20]. The present study demonstrates that MPs released by apoptotic HPCs may carry a death signal to hepatoma-initiating cells. This death signal can reduce the proliferation of hepatoma-initiating cells, thus inhibiting hepatocarcinogenesis.

## Results

### Isolation and characterization of MPs derived from apoptotic HPCs

During apoptosis, cells can release small MPs called apoMPs (< 1 μm in diameter) [21]. In order to obtain a useful amount of apoMPs from apoptotic HPCs, we used different lethal doses of doxorubicin (Additional file 2: Fig. S1A) to treat HPCs (WB-F344 cell line) and the released MPs were then isolated by centrifugation steps (Additional file 2: Fig. S1B) [22]. It has previously been shown that when tumor cells were treated with doxorubicin, the doxorubicin was packaged into the released MPs, and because of the fluorescent nature of doxorubicin, MPs encapsulating doxorubicin could be clearly observed by fluorescence microscopy [22]. The released apoMPs were also observed by fluorescence microscopy in our study. We found that WB-F344 cells treated with 100 µg/ml of doxorubicin released a considerable amount of apoMPs (apoHPC-MPs) (Additional file 2: Fig. S1C). Flow cytometry further showed that 2 × 10^7^ WB-F344 cells released about 1 × 10^6^ apoHPC-MPs (Additional file 2: Fig. S2A, S2B). These apoHPC-MPs had a membrane structure and were ~800 nm in size, as detected by transmission electron microscopy (TEM; Additional file 2: Fig. S2C). The sizes were confirmed by dynamic light scattering (DLS) analysis (Additional file 2: Fig. S2D). DLS analysis also revealed that apoHPC-MPs displayed zeta potentials of ~ − 17 mV (Additional file 2: Fig. S2E). Thus, during apoptosis, HPCs can release large numbers of apoHPC-MPs.

### MPs derived from apoptotic HPCs prevent hepatocarcinogenesis in a primary rat HCC model

**Fig. 1:**
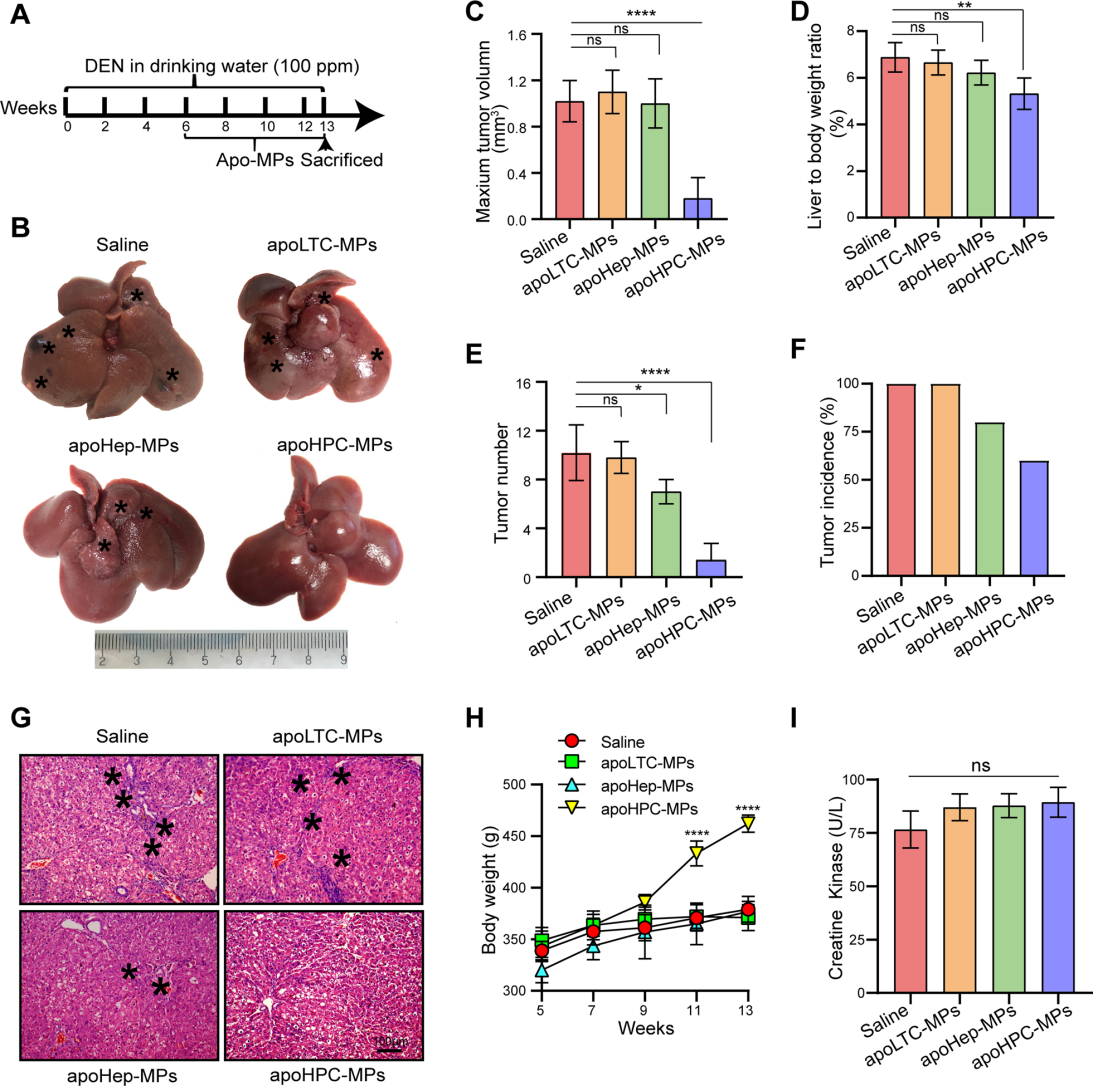
MPS derived from apoptotic HPCs prevent hepatocarcinogenesis in a primary rat HCC model. **A **Diagram of the treatment schedule in the rat HCC model. After 6 weeks of oral treatment with diethylnitrosamine (DEN), Sprague Dawley rats were intrasplenically injected with 40 µg of apoHPC-MPs, apoLTC-MPs or apoHep-MPs in 200 µl saline or 200 µl of blank saline. Injections were administered twice every week for 7 weeks. Oral DEN treatment was also continued during this time. After 13 weeks, the rats were sacrificed to observe the development of hepatocellular carcinoma (HCC). **B** Representative images of rat livers from the indicated groups. Typical tumor nodes are marked by the asterisks. **C** The maximum tumor volume per liver in the different groups. ****p < 0.0001. **D** The liver-to-body weight ratio in the different treatment groups. **p < 0.01. **E** The number of HCC nodules per liver in each group. *p < 0.05, ****p < 0.0001. **F** The tumor incidence in each group. **G** Images of H&E-stained liver sections showing the histological structure and inflammatory response in the indicated groups. Black asterisks represent accumulation of inflammatory cells. (H) Body weight curves. The rats were weighed every other week (n = 5 per group). ****p < 0.0001 compared to saline group. (I) Serological analysis of creatine kinase (CK) was performed after the rats were sacrificed. Data are presented as mean ± SD. ns, not statistically significant

To test whether apoMPs can inhibit hepatocarcinogenesis, it is essential to identify which cellular types are suitable sources of apoMPs. Besides the apoHPC-MPs described above, which were produced from WB-F344 cells, we also produced apoMPs from the hepatocyte cell line BRL (apoHep-MPs) and the liver tumor cell line RH35 (apoLTC-MPs) by treatment with 100 µg/ml of doxorubicin. The apo-MPs were observed by fluorescence microscopy (Additional file 2: Fig. S2F). ApoHPC-MPs, apoHep-MPs and apoLTC-MPs had a similar irregularly spherical morphology as observed by TEM (Additional file 2: Fig. S2C). They also had similar diameters of ~800 nm (Additional file 2: Fig. S2D) and similar zeta potentials of ~ − 17 mV (Additional file 2: Fig. S2E). To test whether these apoMPs have similar anticancer effects, we employed the diethylnitrosamine (DEN)-induced primary HCC model in Sprague Dawley rats. 6 weeks of oral DEN treatment showed the obvious HPC activation (Additional file 2: Fig. S3), HPCs were labeled with an antibody against CK7 [23], and in our previous studies, we found that the activation and malignant transformation of HPCs promote hepatocarcinogenesis and HCC recurrence [9–12]. Thus, the rats were intrasplenically administered with 40 µg of apoLTC-MPs, apoHep-MPs, apoHPC-MPs or saline after 6 weeks of oral DEN treatment. Rats were injected with MPs or saline twice per week for 7 weeks, and oral DEN treatment was continued at the same time. On week 13 of DEN treatment, the rats were sacrificed (Fig. 1A). We then assessed the therapeutic efficacy of apoMPs in the rat HCC primary model. Compared with saline control, apoLTC-MPs or apoHep-MPs, the apoHPC-MPs effectively inhibited tumorigenesis in DEN-exposed rats (Fig. 1B). This was also evidenced by the maximum tumor volume (Fig. 1C), liver-to-body weight ratio (Fig. 1D), tumor number (Fig. 1E) and tumor incidence (Fig. 1F). H&E staining showed that livers from rats treated with apoHPC-MPs had a reduced inflammatory response and a clear tissue structure (Fig. 1G). Besides, we found that apoHPC-MPs ameliorated the weight loss of rats during hepatocarcinogenesis (Fig. 1H). Because of the known toxicity of doxorubicin in heart [22], we also tested whether the apoMPs have side effects on heart. Notably, we did not observe toxic effects of apoHPC-MPs in heart, as shown by serological analysis (creatine kinase) and H&E staining (Fig. 1I and Additional file 2: Fig. S4). ApoHPC-MPs also had no side effects in other major organs, as evidenced by H&E staining of other tissues (Additional file 2: Fig. S4). Taken together, these results show that apoHPC-MPs have much better anticancer efficacy than apoLTC-MPs and apoHep-MPs without typical side effects in a rat primary HCC model.

**Fig. 2:**
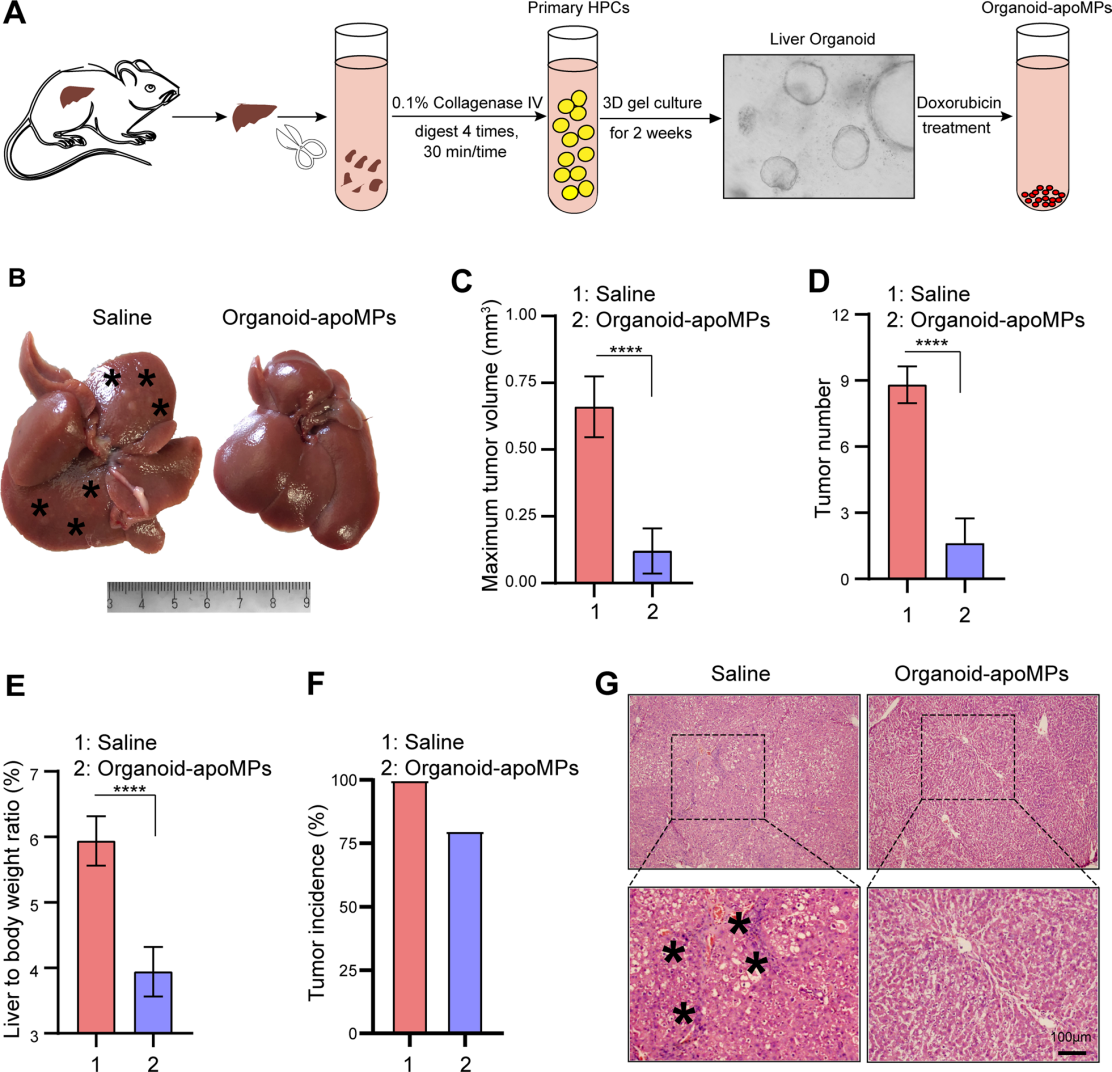
MPs derived from apoptotic primary HPCs prevent hepatocarcinogenesis. **A** Experimental outline for producing organoid-apoMPs. Rats were treated with DEN for 6 weeks. Livers were removed, cut into small pieces, and digested with 0.1% collagenase IV. Primary HPCs were then isolated and cultured to form organoids. 100 µg/ml of doxorubicin was used to treat the organoids and organoid-apoMPs were then isolated. **B** After 6 weeks of DEN treatment, Sprague Dawley rats were intrasplenically injected with 40 µg of organoid-apoMPs in 200 µl saline or 200 µl of blank saline. Injections were given twice every week for 7 weeks. DEN treatment was also continued during this time. After 13 weeks, the rats were sacrificed to observe the development of HCC. Representative images of livers are shown. Typical tumor nodes are indicated by the asterisks. **C** The maximum tumor volume per liver in the two groups. Data are presented as mean ± SD. ****p < 0.0001. **D** The number of HCC nodules per liver. Data are presented as mean ± SD. ****p < 0.0001. **E** The liver-to-body weight ratio. Data are presented as mean ± SD. ****p < 0.0001. **F** The tumor incidence in the two groups. **G** Images of H&E-stained liver sections, showing the histological structure and inflammatory response of the liver in the indicated groups. Black asterisks represent accumulation of inflammatory cells

In order to further confirm the role of apoHPC-MPs on hepatocarcinogenesis, we isolated primary HPCs from rats via collagen IV digestion and formed liver organoids. The organoids were treated with 100 µg/ml of doxorubicin, then organoid-apoMPs were produced (Fig. 2A). In order to test the therapeutic efficacy of organoid-apoMPs, the rat primary HCC model was established and the animals were intrasplenically treated with organoid-apoMPs or saline. Organoid-apoMPs (40 µg) were administered to rats after 6 weeks of DEN treatment to induce hepatocarcinogenesis. Rats were treated with MPs or saline twice per week for 7 weeks. On week 13 of DEN treatment, the rats were sacrificed and the tumor growth was evaluated. As shown in Fig. 2B, almost no tumor nodes were observed in the organoid-apoMPs treatment group. In contrast, several tumor nodes were observed in the saline control group. Treatment with organoid-apoMPs also reduced the maximum tumor volume (Fig. 2C), tumor number (Fig. 2D), liver-to-body weight ratio (Fig. 2E) and tumor incidence (Fig. 2F). Compared with livers from the saline group, livers from the organoid-apoMPs group showed a reduced inflammatory response and a clear tissue structure, as revealed by H&E staining (Fig. 2G). Therefore, primary HPC-derived apoMPs efficiently inhibit hepatocarcinogenesis.

### HPCs take up more apoHPC-MPs than apoLTC-MPs and apoHep-MPs

**Fig. 3:**
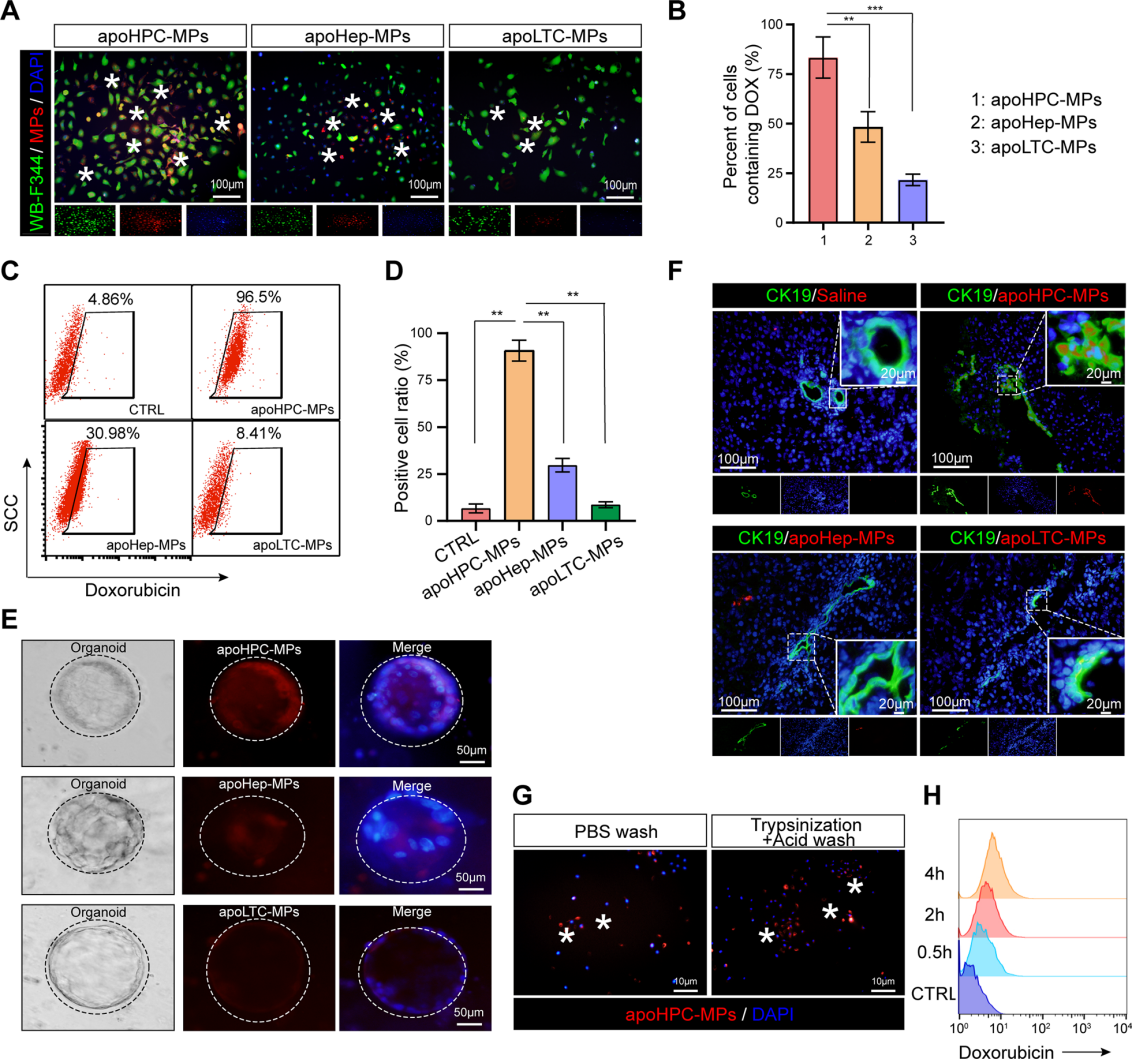
HPCs take up more apoHPC-MPs than apoLTC-MPs or apoHep-MPs. **A**, **B** WB-F344 cell (5 × 10^3^) were labeled with GFP (green fluorescence), incubated for 30 min with 1 × 10^5^ apoHPC-MPs, apoLTC-MPs or apoHep-MPs containing DOX (red fluorescence). The uptake of MPs into WB-F344 cells was observed by fluorescence microscopy. Representative images are shown in **A**. The percentage of WB-F344 cells with red fluorescence (indicating uptake of MPs) was calculated in each group. The combined data from three experiments are shown in **B**. Data are presented as mean ± SD. **p < 0.01, ***p < 0.001. **C**, **D** 1 × 10^6^ ApoHPC-MPs, apoLTC-MPs and apoHep-MPs containing doxorubicin (red fluorescence) were incubated with WB-F344 cells (3 × 10^5^) for 30 min. The percentage of WB-F344 cells containing red fluorescence was measured by flow cytometry. Representative flow cytometry data are shown in **C**. The combined data from three experiments are shown in (D). Data are presented as mean ± SD. **p < 0.01. **E** Liver organoids were cultured in vitro. 1 × 10^4^ apoHPC-MPs, apoLTC-MPs or apoHep-MPs were incubated with liver organoids for 30 min. The uptake of MPs into organoids was observed by fluorescence microscopy. Representative images are shown. Nuclei were stained with DAPI (blue). **F** 1 × 10^7^ apoHPC-MPs, apoHep-MPs or apoLTC-MPs were intrasplenically injected into DEN-treated rats. 30 min after injection, liver slices were acquired for fluorescence detection. HPCs were recognized by antibodies against CK19 (green). Nuclei were stained with DAPI (blue). HPCs efficiently took up red fluorescent apoHPC-MPs. **G** WB-F344 cells (3 × 10^5^) were incubated with apoHPC-MPs (1 × 10^5^) for 30 min. Then WB-F344 cells were treated with 0.2 × trypsin/EDTA buffer for 1 min and then washed with citric acid buffer several times to remove all non-internalized apoHPC-MPs bound to the surface of WB-F344 cells. Nuclei were stained with DAPI (blue). **H** 3 × 10^5^ WB-F344 cells were incubated with 2 × 10^5^ apoHPC-MPs for 0.5, 2, and 4 h. After washing, the samples were subjected to flow cytometric analysis. A representative image is shown here

The above results show that apoHPC-MPs prevent hepatocarcinogenesis more efficiently than apoLTC-MPs and apoHep-MPs. To elucidate the underlying mechanism, we first asked whether HPCs preferntially take up apoHPC-MPs compared to apoLTC-MPs and apoHep-MPs. Therefore, doxorubicin (100 µg/ml) was added to cultured WB-F344 cells (1 × 10^7^), RH35 cells (1 × 10^7^) or BRL cells (1 × 10^7^). After 12 h, the released apoHPC-MPs, apoLTC-MPs and apoHep-MPs were isolated from the culture medium by centrifugation. The same numbers of apoHPC-MPs (1 × 10^5^), apoLTC-MPs (1 × 10^5^) and apoHep-MPs (1 × 10^5^) were incubated with WB-F344 cells (5 × 10^3^) for 30 min. Interesting, only the apoHPC-MPs, and not the apoLTC-MPs and apoHep-MPs, were efficiently taken up by WB-F344 cells (Fig. 3A). Around 75% of WB-F344 cells took up apoHPC-MPs (Fig. 3B). Furthermore, the specific targeting of apoHPC-MPs, apoLTC-MPs and apoHep-MPs was quantitatively determined by flow cytometry analysis. Equivalent numbers of apoHPC-MPs (1 × 10^6^), apoLTC-MPs (1 × 10^6^) and apoHep-MPs (1 × 10^6^) were incubated with WB-F344 cells (3 × 10^5^) for 30 min. The results indicated that 96% of WB-F344 cells were positive for apoHPC-MPs, whereas only about 8% of WB-F344 cells were positive for apoLTC-MPs, and about 30% of WB-F344 cells were positive apoHep-MPs (Fig. 3C, D). This suggests that WB-F344 cells selectively take up apoHPC-MPs, rather than apoLTC-MPs or apoHep-MPs. Consistently, RH35 cells showed a strong preference for apoLTC-MPs, rather than apoHPC-MPs and apoHep-MPs (Additional file 2: Fig. S5A). Based on flow cytometry analysis, about 97% of RH35 cells were positive for apoLTC-MPs, while only about 12% of RH35 cells took up apoHep-MPs and about 38% of RH35 cells took up apoHPC-MPs (Additional file 2: Fig. S5B). BRL cells also indicated a consistent pattern of uptake, with a strong preference for apoHep-MPs, rather than apoHPC-MPs and apoLTC-MPs (Additional file 2: Fig. S5C, D). These results indicate that cells can efficiently take up parental cell-derived MPs. Thus, apoHPC-MPs efficiently target WB-F344 cells.

To further test the targeting of apoMPs to HPCs, we isolated primary HPCs from rat liver and generated organoids. Equivalent numbers of apoHPC-MPs (1 × 10^4^), apoLTC-MPs (1 × 10^4^) and apoHep-MPs (1 × 10^4^) were incubated with primary HPCs for 30 min. Primary HPCs efficiently took up apoHPC-MPs, rather than apoHep-MPs and apoLTC-MPs, as evidenced by fluorescence microscopy (Fig. 3E). Because all these findings were based on cultured HPCs, we wanted to further clarify whether apoMPs have the same ability to target HPCs in vivo. For this experiment, 1 × 10^7^ of apoHPC-MPs, apoHep-MPs and apoLTC-MPs were intrasplenically injected into DEN-treated rats and liver slices were acquired for fluorescence detection 30 min after injection. The expression of the HPC marker CK19 in liver sections was examined [24–26]. As observed in cell culture, accumulation of red fluorescent apoHPC-MPs, rather than apoHep-MPs and apoLTC-MPs, was found in CK19-positive HPCs (Fig. 3F). This demonstrates that HPCs can selectively take up HPC-derived apoHPC-MPs in vivo.

We also examined the interaction of HPCs and apoHPC-MPs. WB-F344 cells (3 × 10^5^) were incubated with apoHPC-MPs (1 × 10^5^) for 30 min, then treated with trypsin and washed several times with citric acid to remove the surface-bound MPs. Fluorescence imaging revealed that apoHPC-MPs were not cleared from WB-F344 cells after trypsinization and acid washing (Fig. 3G). This suggests that apoHPC-MPs were internalized into WB-F344 cells, rather than being attached to the surface of the WB-F344 cells. We further studied the dynamics of apoHPC-MPs internalization into WB-F344 cells. For this experiment, 3 × 10^5^ WB-F344 cells were incubated with 2 × 10^5^ apoHPC-MPs. The internalization of apoHPC-MPs was detected after 0.5 h and increased markedly with time (Fig. 3H), suggesting that the uptake of apoHPC-MPs by HPCs is time-dependent.

### ApoHPC-MPs are cytotoxic to HPCs

**Fig. 4:**
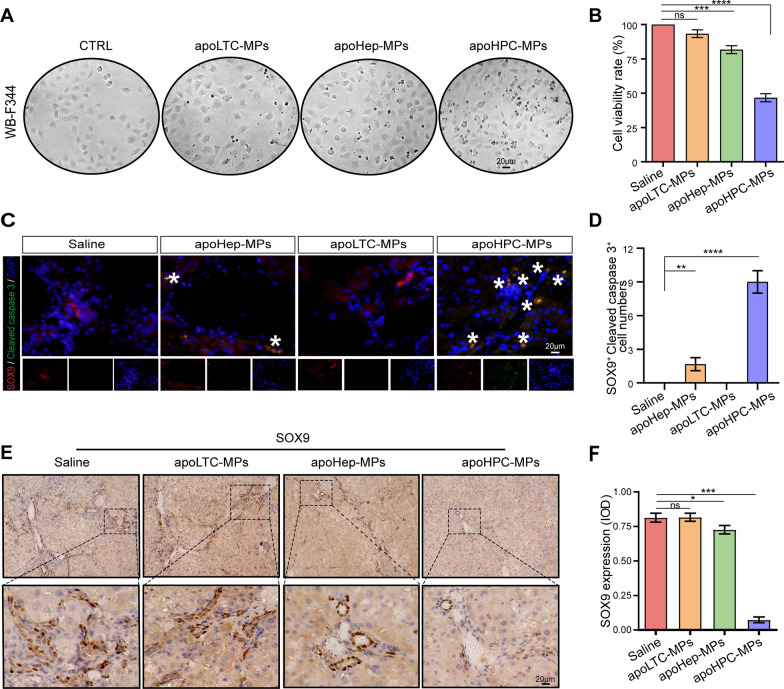
ApoHPC-MPs are cytotoxic to HPCs. **A** The same amount (20 µg) of apoLTC-MPs, apoHep-MPs or apoHPC-MPs was incubated with cultured WB-F344 cells (5 × 10^3^) for 24 h. The killing of WB-F344 cells was observed by microscopy. **B** WB-F344 cells were treated with 20 µg of apoHPC-MPs, apoHep-MPs or apoLTC-MPs for 1 h. The cells were washed and cultured in fresh medium for 24 h. Cell proliferation was detected by CCK8 assay. Data were collected from three independent experiments. Data are presented as mean ± SD. ***p < 0.001, ****p < 0.0001. **C** 40 µg of apoHPC-MPs, apoHep-MPs or apoLTC-MPs were intrasplenically injected into rats, which were treated with DEN for the previous 6 weeks. 24 h after injection, liver slices were acquired for fluorescence detection. HPCs were stained with antibodies against SOX9 (red) and cleaved caspase 3 (green) for fluorescence microscopy analysis. Nuclei were stained with DAPI (blue). Representative pictures of staining are shown. White asterisks show the SOX9-positive HPCs which are also positive for cleaved caspase 3. **D** Cells positive for both SOX9 and cleaved caspase 3 were counted in each field of view in **C**; the combined data from three experiments are shown. **p < 0.01, ****p < 0.0001 **E** 40 µg of apoHPC-MPs, apoHep-MPs or apoLTC-MPs were intrasplenically injected into rats, which were treated with DEN for the previous 6 weeks. Injections were administered twice every week for 4 weeks. Rats were then sacrificed, and liver tissue sections were acquired for IHC assay. The HPCs were stained with an antibody against SOX9. Representative photographs are shown. In the apoHPC-MP-treated liver, SOX9 staining is faint and very scarce. The apoHep-MP-treated liver shows a moderate level of SOX9 staining. The other livers show strong and diffuse staining. **F** Computer-assisted IOD evaluation of SOX9 staining in **E**. Data were collected from three independent experiments. Data are presented as mean ± SD. *p < 0.05, ***p < 0.001

Next, we asked if apoHPC-MPs are cytotoxic to HPCs after they are internalized. ApoHPC-MPs (20 µg), apoLTC-MPs (20 µg) or apoHep-MPs (20 µg) were added to cultured WB-F344 cells (5 × 10^3^) for 24 h. Compared with apoHep-MPs and apoLTC-MPs, apoHPC-MPs induced more killing of WB-F344 cells (Fig. 4A). Moreover, the proliferation of WB-F344 cells was efficiently inhibited by treatment with apoHPC-MPs (Fig. 4B). To test this result *in vivo*, 40 µg of apoHPC-MPs, apoHep-MPs or apoLTC-MPs were intrasplenically injected into rats that previously received DEN treatment for 6 weeks. After 24 h, liver slices were acquired for fluorescence detection. HPCs were labeled with an antibody against SOX9 [26, 27] and apoptotic cells were detected with an antibody against cleaved caspase 3. The highest number of apoptotic HPCs (positive for both SOX9 and cleaved caspase 3) was seen in the apoHPC-MPs treatment group (Fig. 4C, D). In the apoHep-MPs group, a small number of HPCs were positive for cleaved caspase 3 staining, while saline and apoLTC-MPs did not induce apoptosis of HPCs (Fig. 4C, D). To corroborate these observations, we also evaluated the expression of the HPC marker SOX9 and CK7 in liver sections by IHC [23, 24]. ApoLTC-MPs and the control group showed intense and diffuse positive SOX9 and CK7 staining, while apoHep-MPs caused a small decrease in the SOX9 and CK7 signal and apoHPC-MPs caused a significant decrease in SOX9 and CK7 staining (Fig. 4E, F and Additional file 2: Fig. S6). Therefore, after injection of apoMPs into rats, the number of HPCs was greatly reduced by apoHPC-MPs, partly reduced by apoHep-MPs, and unaffected by apoLTC-MPs. As a result of all these experiments, we conclude that HPCs can selectively take up cytotoxic apoHPC-MPs, inhibiting the proliferation of hepatoma-initiating cells.

### MPs from non-apoptotic HPCs have no affect hepatocarcinogenesis

**Fig. 5:**
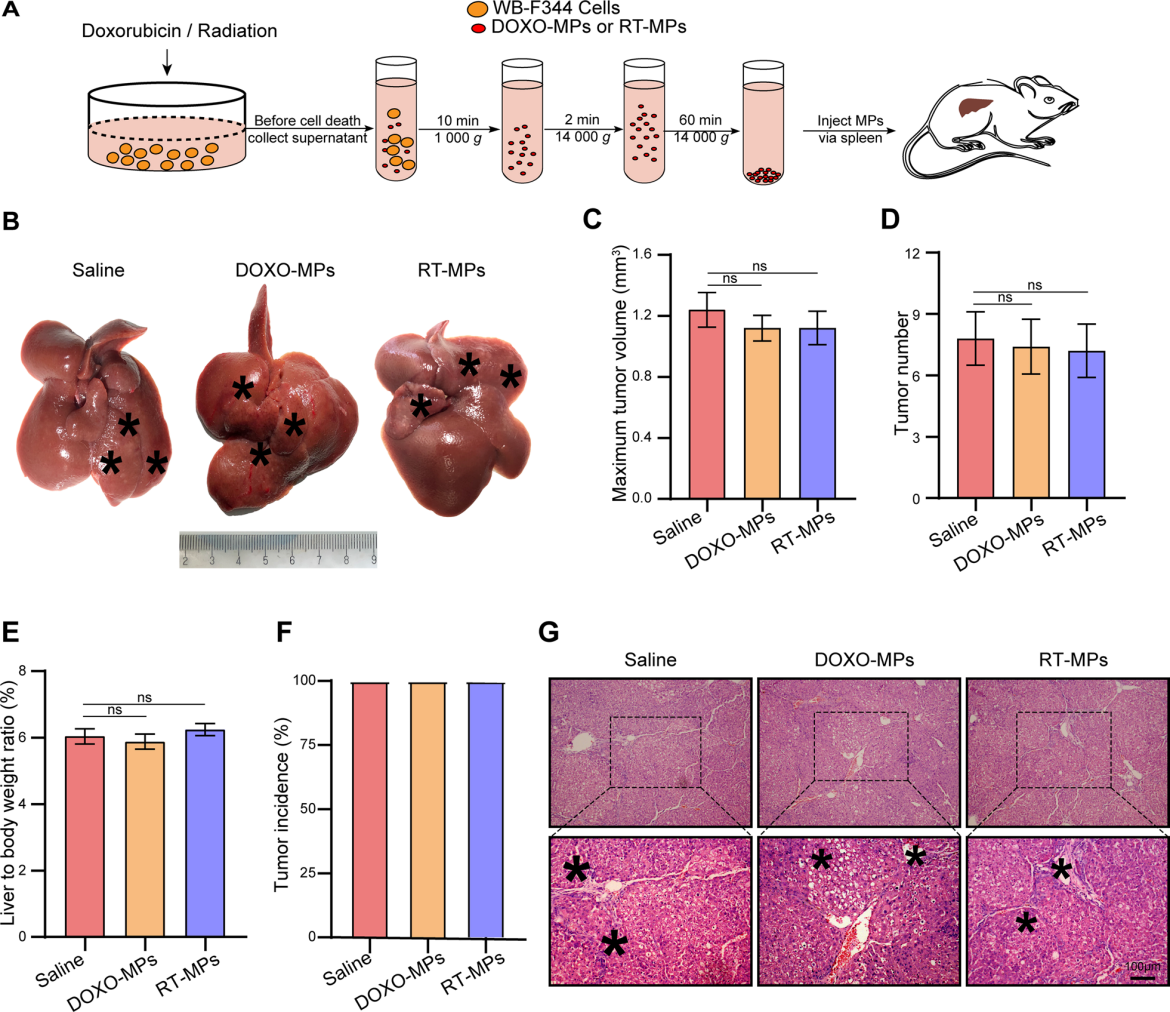
HPC-derived MPs without death signals have no effect on hepatocarcinogenesis. **A** Experimental outline for producing MPs without apoptotic signals (DOXO-MPs and RT-MPs). WB-F344 cells were treated with 100 µg/ml doxorubicin or 20 Gy radiation. DOXO-MPs and RT-MPs were isolated before the cells underwent apoptosis. **B**–**F** Sprague Dawley rats, which were treated with DEN for 6 weeks, were intrasplenically injected with 40 µg of DOXO-MPs and RT-MPs. Injections were administered twice every week for 7 weeks. DEN treatment was also continued during this time. After 13 weeks, the rats were sacrificed to observe the development of hepatocellular carcinoma (HCC). Representative images of rat livers are shown. Typical tumor nodes are marked by the asterisks (**B**). The maximum tumor volume per liver in the indicated groups (**C**). The number of HCC nodules per liver (**D**). The liver-to-body weight ratio (**E**). The tumor incidence in the different treatment groups (**F**). **G** Images of H&E-stained liver sections showing the histological structure and inflammatory response after the indicated treatments. Black asterisks indicate accumulation of inflammatory cells

To further clarify the role of the death signal, we generated MPs from non-apoptotic HPCs. WB-F344 cells were treated with 100 µg/ml of DOX, or irradiated with 20 Gy, and then MPs without death signal (DOXO-MPs and RT-MPs) were collected from the cells before they underwent apoptosis. 40 µg of DOXO-MPs and RT-MPs were injected into rats, which were treated with DEN for 6 weeks (Fig. 5A). MPs or saline were injected twice every week for 7 weeks. The rats were then sacrificed to observe the tumor growth. As shown in Fig. 5B, compared with the saline control group, treatment with DOXO-MPs and RT-MPs had no effect on hepatocarcinogenesis. This result was also confirmed by the maximum tumor volume (Fig. 5C), tumor number (Fig. 5D), liver-to-body weight ratio (Fig. 5E) and tumor incidence (Fig. 5F). H&E staining showed that the inflammatory response was similar in the livers of each group (Fig. 5G). Therefore, MPs derived from non-apoptotic HPCs had no effect on hepatocarcinogenesis.

Because apoHPC-MPs were produced by treating HPCs with doxorubicin, the released apoHPC-MPs contained doxorubicin in addition to the death signal (Additional file 2: Fig. S1C). To exclude any effect of the therapeutic drug doxorubicin on killing HPCs by apoHPC-MPs in the rat primary HCC model, the amount of doxorubicin in apoHPC-MPs was detected by liquid chromatography-mass spectrometry (LC-MS) analysis (Additional file 2: Fig. S7A). Free doxorubicin (~ 10 µg, which was the same amount of doxorubicin encapsulated in apoHPC-MPs for injection of each rat) was intrasplenically injected into rats, which had been treated with DEN for 6 weeks. Injections were performed twice every week for 7 weeks. The rats were then sacrificed for tumor examination. Compared with the saline control group, the free doxorubicin had no significant effect on hepatocarcinogenesis (Additional file 2: Fig. S7B). There was no significant difference in the maximum tumor volume (Additional file 2: Fig. S7C), tumor number (Additional file 2: Fig. S7D), liver-to-body weight ratio (Additional file 2: Fig. S7E) and tumor incidence (Additional file 2: Fig. S7F). H&E staining showed that the inflammatory response was similar in the livers of each group (Additional file 2: Fig. S7G). Therefore, doxorubicin encapsulated in apoHPC-MPs had no effect on hepatocarcinogenesis.

### HPC-derived MPs containing death signals inhibit hepatocarcinogenesis in the absence of doxorubicin

**Fig. 6:**
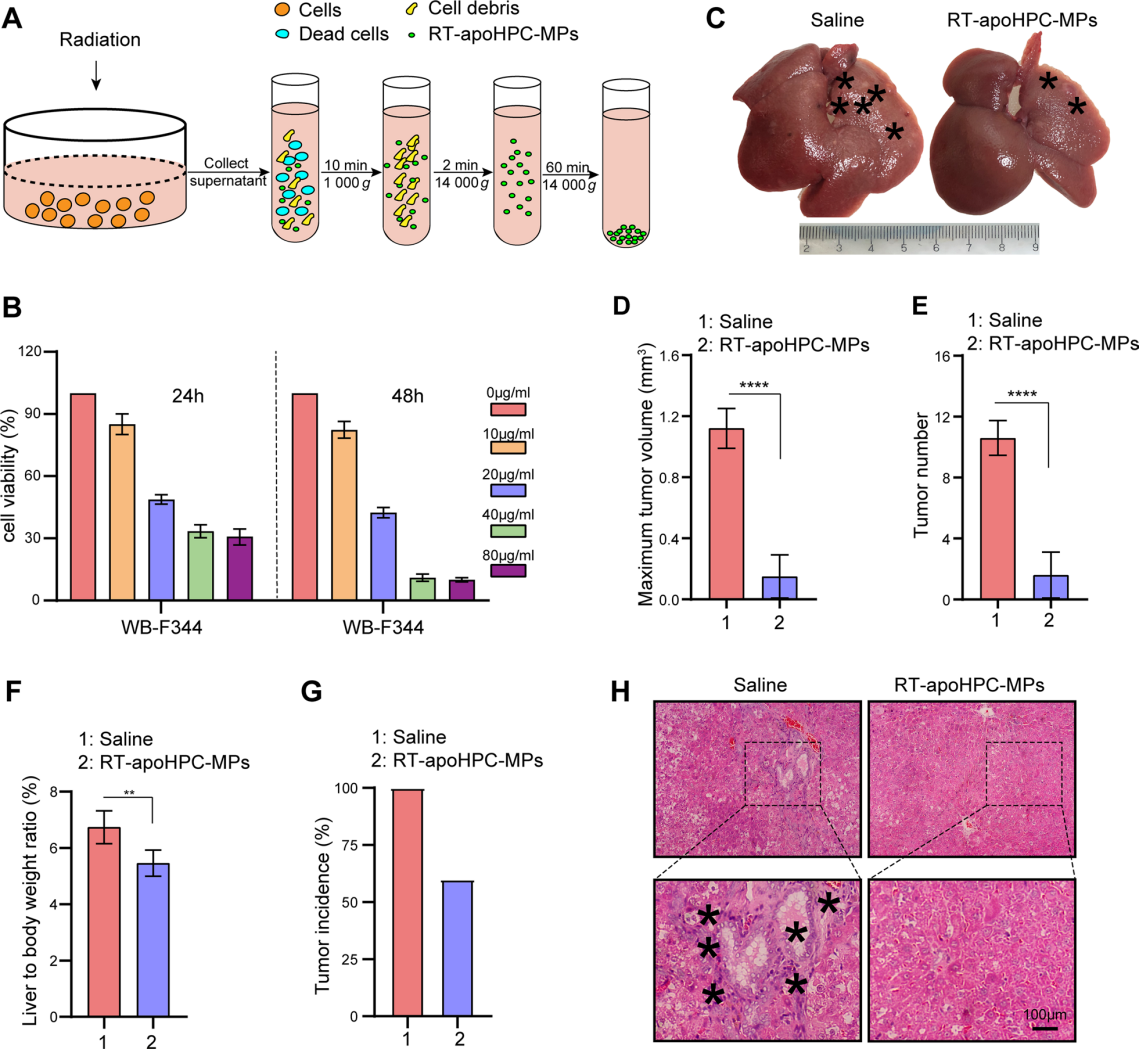
HPC-derived MPs carrying the death signal inhibit hepatocarcinogenesis in the absence of doxorubicin. **A** Experimental outline for producing RT-apoHPC-MPs. WB-F344 cells were treated with 20 Gy of radiation and RT-apoHPC-MPs were isolated after apoptosis of the cells. **B** WB-F344 cells were treated with various concentrations of RT-apoHPC-MPs for 24 or 48 h, and cell viability was detected using the CCK-8 assay. **C**–**G** Rats were treated with DEN for 6 weeks, then intrasplenically injected with RT-apoHPC-MPs (40 µg). Injections were administered twice every week for 7 weeks. DEN was administered orally in parallel. After 13 weeks, the rats were sacrificed to observe the development of HCC. Representative images of rat livers are shown. Typical tumor nodes are marked by the asterisks (**C**). The maximum tumor volume per liver in the two groups (**D**). The number of HCC nodules per liver (**E**). The liver-to-body weight ratio in the two groups (**F**). The tumor incidence in the two groups (**G**). Data are presented as mean ± SD. **p  < 0.01, ****p < 0.0001. **H** Images of H&E-stained liver sections showing the histological structure and inflammatory response of liver in the two groups. Black asterisks indicate accumulation of inflammatory cells

To further clarify the role of the death signal in MPs, and exclude the effect of the encapsulated chemotherapeutic drug, we wanted to isolate MPs without chemotherapeutic drugs. To do this, we used a lethal dose of radiation to induce WB-F344 cell death. WB-F344 cells were treated with various doses of radiation to establish the lethal dose. We observed increased cell killing with an increased radiation dose until 20 Gy, which induced the maximum level of cell death (Additional file 2: Fig. S8A). The amount of MPs differed according to the subsequent culture time of cells after 20 Gy radiation: the maximal amount of MPs was isolated after culturing WB-F344 cells for another 72 h (Additional file 2: Fig. S8B). We chose these conditions for subsequent studies. The MPs were designated as RT-apoHPC-MPs (Fig. 6A). To further evaluate the therapeutic effect of RT-apoHPC-MPs on HPCs, we performed cell toxicity studies on the HPC cell line WB-F344. We found that RT-apoHPC-MPs efficiently inhibited the growth of WB-F344 cells in a dose- and time-dependent manner (Fig. 6B and Additional file 2: Fig. S8C). We then investigated the therapeutic effect of RT-apoHPC-MPs in the rat primary HCC model. RT-apoHPC-MPs (40 µg) were intrasplenically injected into rats, which had been treated with DEN for 6 weeks. Injections were performed twice every week for 7 weeks. The rats were then sacrificed for tumor examination. As shown in Fig. 6C, we observed reduced tumor growth upon treatment with RT-apoHPC-MPs, superior to that of the control treatment. This result was supported by analysis of maximum tumor volume (Fig. 6D), tumor number (Fig. 6E), liver-to-body weight ratio (Fig. 6F) and tumor incidence (Fig. 6G). H&E staining showed a reduced inflammatory response and clear tissue structure in the livers of rats treated with RT-apoHPC-MPs (Fig. 6H). Therefore, RT-apoHPC-MPs carrying a death signal can efficiently inhibit hepatocarcinogenesis in the absence of doxorubicin.

### Quantitative proteomic analysis of extracellular microparticles reveals enrichment of death-related proteins in apoHPC-MPs

**Fig. 7:**
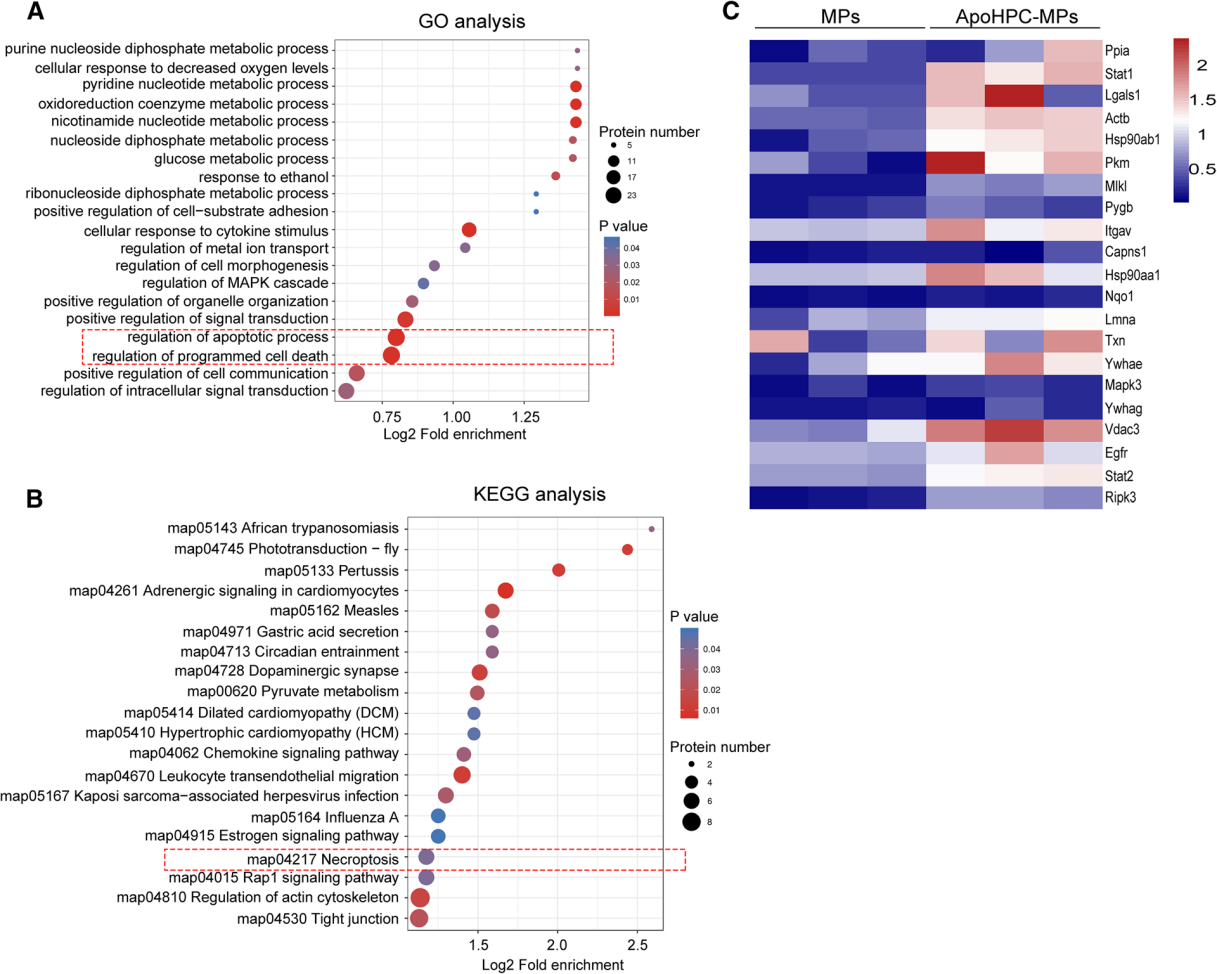
Quantitative proteomic analysis of extracellular microparticles reveals enrichment of death related proteins in apoHPC-MPs. **A** Differentially expressed (up-regulated) proteins between apoHPC-MPs and MPs were classified by GO annotation based on the following category: biological process. The bubble chart shows the top 20 categories with the most significant enrichment. The vertical axis shows the function classification. The x-axis indicates Log2 of the ratio of the number of differential proteins in the corresponding function classification to the number of total proteins identified. The colors of the points represent the P values of enrichment significance. The sizes of the dots represent the numbers of differential proteins in the corresponding function classification. **B** Annotation of differentially expressed (up-regulated) proteins between apoHPC-MPs and MPs based on the KEGG pathway database. The bubble chart shows the top 20 categories with the most significant enrichment. The vertical axis shows the functional pathway. The x-axis indicates Log2 of the ratio of the number of differential proteins in the corresponding pathway to the number of total proteins identified. The colors of the points represent the P values of enrichment significance. The sizes of the dots represent the numbers of differential proteins in the corresponding pathway. **C** Based on the MS results, heat map of the up-regulated gene encoding death-related proteins in apoHPC-MPs compared to MPs

To further investigate the death signal encapsulated by apoHPC-MPs, quantitative proteomics analysis was performed on apoHPC-MPs from apoptotic WB-F344 cells and MPs from non-apoptotic WB-F344 cells by MS/MS. Three independent experiments reliably identified 523, 344 and 652 proteins from MPs, and 310, 769 and 978 proteins from apoHPC-MPs. Of these, 278 proteins were quantitatively identified in all replicates (Additional file 1: Table S1). The threshold for significant up-regulation was set as fold change > 1.5 and the threshold for significant down-regulation was set as less than 1/1.5. Under these criteria, there were 116 proteins significantly upregulated in apoHPC-MPs as compared to MPs (Additional file 1: Table S1). Gene ontology (GO) enrichment analysis indicated that the 116 up-regulated proteins were enriched in biological processes related to regulation of apoptotic process and regulation of programmed cell death (Fig. 7A). Further, Kyoto Encyclopedia of Genes and Genomes (KEGG) Analysis showed that the 116 up-regulated proteins were also related to the necroptosis pathway (Fig. 7B). Moreover, based on 116 up-regulated proteins, gene encoding death-related proteins are present at higher levels in the apoHPC-MPs than in MPs (Fig. 7C). Together, these results indicate that apoHPC-MPs inhibit hepatocarcinogenesis by transmitting a death signal to hepatoma-initiating cells, which results in the death of hepatoma-initiating cells.

## Discussion

There are several major reasons for choosing MPs as vehicles for therapeutic reagents to prevent hepatocarcinogenesis. Firstly, MPs have intrinsically fewer adverse effects compared to other artificial delivery vehicles such as liposomes, and MPs can carry bioactive molecules into targeted cells. Thus, we hypothesize that MPs containing a death signal can kill the recipient cells. Secondly, MPs have specificity in targeting liver tissue. Thirdly, although MPs may be swallowed by mononuclear phagocytosis (Additional file 2: Fig. S9), macrophages also contribute to hepatocarcinogenesis. If MPs carrying a death signal are internalized by macrophages, this may result in the death of tumor-related macrophages, inhibiting the formation of liver tumors. Moreover, there are several major reasons for choosing vesicles derived from hepatic progenitor cells. Firstly, HPC-derived vesicles are harmless; they cannot carry oncogenes and cannot transform recipient cells into malignant tumor cells. However, MPs derived from tumor cells have oncogenic potential. For example, it was found that tumor cell-derived MPs contain oncogene sequences and alter the transcriptome of recipient cells [28]. These MPs also promoted tumor angiogenesis and tumor metastasis [29, 30]. Secondly, MPs may be taken up preferentially by cells that originate from, MPs produced from self-derived HPCs may directly target to the hepatoma-initiating cells in liver tissue. These potential advantages of HPC-derived MPs make them ideal for delivering therapeutic agents to prevent the proliferation of hepatoma-initiating cells.

We then explored the feasibility of delivering the death signal to hepatoma-initiating cells using MPs derived from apoptotic HPCs. Our data show that HPC-derived MPs carrying a death signal can target and inhibit the proliferation of hepatoma-initiating cells in liver, thus preventing hepatocarcinogenesis. This effect has not been described so far for MPs, especially for MPs from HPCs. MPs from HPCs, but not from liver cells or liver tumor cells, can target to HPCs. This targeting phenomenon agrees with other studies. Several reports have verified that extracellular microvesicles can target the original cells. For example, it was found that macrophage-derived MPs can effectively be taken up by immune cells [31]. MPs released by mature dendritic cells (DCs) can selectively target B cells [32]. Tumor cell-derived MPs are capable of delivering chemotherapeutic drugs to tumor cells [22]. There are various possibilities to explain the targeting of MPs to recipient cells. For example, ligands on the surface of extracellular microvesicles may interact with receptors on the surface of recipient cells. MPs derived from endothelial progenitor cells target endothelial cells by interaction with the adhesion molecules integrin alpha 4 and beta 1 on the MP surface [33]. Exosomes are taken up by endothelial and pancreatic cell because exosomes carry the Tspan8-integrin alpha 4 complex which interacts with the CD54 ligand on endothelial and pancreatic cells [34]. Exosome targeting to DCs is mediated via MFG-E8/lactadherin, CD11a, CD54, CD9 and CD81 on the surface of exosomes and integrin alpha V/integrin beta 3, CD11a and CD54 on the surface of DCs [35]. In the current study, we also found that apoHPC-MPs carry proteins which have been reported to mediate the targeting of exosomes, such as integrin alpha V and MFG-E8/lactadherin (Additional file 1: Table S1) [35]. Moreover, our data also identified some adhesion and motility molecules in the proteome of apoHPC-MPs (Additional file 1: Table S1), such as CD44, CD81, fibronectin, CD151 and CD99. Studies indicated that these molecules mediate the homing, adhesion, motility, and migration of cells [36–38]. Therefore, based on the literature and our findings, these molecules may mediate the targeting of apoHPC-MPs to the hepatoma-initiating cells in rat liver.

ApoMPs are generated during cell death. Apoptotic bodies (apoBDs) were the first identified apoMPs [39, 40]. Currently, apoBDs can be distinguished from apoMPs according to their diameter: ApoBDs have diameters of 1000–5000 nm [40, 41] and apoMPs have smaller diameters of 200–1000 nm. ApoMPs carry functional biomolecules to targeted cells. Li et al. and Sarah et al. comprehensively discussed the function of apoMPs in tumor treatment [21, 40]. Currently, the main function of apoMPs in tumor treatment is based on apoMP-initiated antitumor immunity. ApoMPs carry the tumor antigen, which can then be transported to antigen-presenting cells and dendritic cells to induce T cell responses and promote antitumor immunity [40, 42]. It was also reported that macrophages are involved in T cell-mediated antitumor immunity by apoMPs [43]. Our current study broadens the application of apoMPs in tumor treatment. In the current study, we found that apoMPs derived from HPCs can prevent hepatocarcinogenesis. The underlying mechanism is that apoHPC-MPs carrying a death signal are directly taken up by the hepatoma-initiating cells, which are then killed, thus preventing hepatocarcinogenesis. In detail, according to the quantitative proteomics analysis, we found that apoHPC-MPs contained the proteins which were involved in the regulation of cell death. Although apoHPC-MPs contained some anti-apoptotic proteins such as CAPNS1 [44], the pro-apoptotic proteins were also obviously upregulated in apoHPC-MPs such as PKM, STAT1, STAT2, VDAC3, and these proteins induced the apoptosis of cells by different pathway [45–48]. Besides, we also found that MLKL and RIPK3 were obviously upregulated in apopHPC-MPs, the study indicated that MLKL and RIPK3 were involved in the regulation of necroptosis [49]. Thus, we speculate that death signals in apoHPC-MPs may induce the death of hepatoma-initiating cells together.

## Conclusions

In summary, our current study found that compared with apoHep-MPs and apoLTC-MPs, apoHPC-MPs can efficiently inhibit hepatocarcinogenesis in the rat primary HCC model. Further, the hepatoma-initiating cells efficiently take up apoHPC-MPs, but not apoHep-MPs and apoLTC-MPs. Moreover, apoHPC-MPs are biological vectors; the death signal encapsulated in apoHPC-MPs can directly kill the hepatoma-initiating cells. This study may provide a new way to prevent liver cancer.

## Methods

### Cell culture

The rat liver tumor cell line RH35, the rat hepatocyte cell line BRL and the rat hepatic progenitor cell line WB-F344 were purchased from Cell Bank of Type Culture Collection of Chinese Academy of sciences, Shanghai Institute of Cell Biology, Chinese Academy of Sciences. Cell were cultured in DMEM medium (Gibco, Invitrogen, Carlsbad, CA, USA) supplemented with 10% fetal bovine serum (FBS, Gibco, Invitrogen), penicillin and streptomycin. Cells were maintained in 5% CO_2_ and 37 °C in a humidified incubator, and sub-cultured every 2 days when they reached 70–80% confluence.

### Culture and establishment of rat adult liver 3D organoids

Male F344 rats were obtained from Shanghai Laboratory Animal Center (Shanghai, China), and were housed in a pathogen-free animal facility. All animals received human care in accordance with the National Institutes of Health guide. The rats were 8–10 weeks old and had body weights of 160 g ± 20 g. The experimental procedures were approved by the Institutional Lab Animal Care and Use Committee.

Rats were fed with 0.01% concentration of DEN in drinking water. Primary HPCs were isolated from SD rats after 6 weeks of treatment with diethylnitrosamine (DEN) at 0.01% concentration. Rats were anesthetized with pentobarbital sodium (30 mg/kg). The liver was removed by surgical excision, then kept cold at 4 °C in basal medium in a 100-mm petri dish. The liver was minced into pieces of roughly 0.5 mm^3^ using fine scissors and the tissue pieces were washed. 10 ml of digestion solution (0.1% of type IV collagenase) prewarmed to 37 °C were added, and the digestion mixture was incubated on a shaker at 37 °C. When the ductal structure appeared, the supernatant was transferred to a fresh 15 ml centrifuge tube. The supernatant was filtered through 70 and 40 μm mesh filters. The cells were collected and suspended in DMEM medium, centrifuged at 290*g* for 5 min, then mixed with matrigel, and seeded in plates. Then the cells were cultured in advanced DMEM supplemented with epidermal growth factor (EGF), hepatocyte growth factor (HGF) and other factors.

### Generation and isolation of apoptotic MPs (apo-MPs)

Cells were treated with the chemotherapeutic drug doxorubicin or a lethal dose of radiation followed by incubation at 37 °C. Doxorubicin was purchased from Shanxi Pude Pharmaceutical Co., Ltd. (Shanxi, China). The rate of apoptosis was detected by flow cytometry using V-fluorescein isothiocyanate (FITC) Annexin (BD Bioscience). At the time of MP harvest, at least 95% of cells should be apoptotic. Apoptotic cells were washed and added to serum-free medium. MPs released by the apoptotic cells were purified from the culture supernatant. The supernatant was first centrifuged at 1000*g* for 10 min and 14,000*g* for 2 min to remove remaining cells and debris, and then subsequently centrifuged at 14,000*g* for 60 min to isolate MPs, which were washed at 14,000*g* for 40 min. Isolated vesicles were then used for assays as indicated in the text.

### Quantification of apo-MPs

Apo-MPs were quantified by measuring the protein concentration with the BCA Protein Assay Kit (Thermo Fisher Scientific) according to the manufacturer’s protocol. In detail, apoMPs were lysed with radioimmunoprecipitation assay (RIPA) buffer at 4 °C for 30 min, then centrifuged for 10 min at 12,000*g* at 4 °C. The supernatant was then collected and protein was quantified.

### Animal model and treatment with apo-MPs

One hundred male F344 rats were obtained from Shanghai Laboratory Animal Center (Shanghai, China), and were housed in a pathogen-free animal facility. All animals received human care in accordance with the National Institutes of Health guide. The rats were 8–10 weeks old and had body weights of 160 g ± 20 g. Rats were fed with 0.01% concentration of DEN in drinking water. After 6 weeks, apoptotic cell-derived MPs were injected into the spleen of the DEN-pretreated rats. Rats were anesthetized with pentobarbital sodium (30 mg/kg). In detail, to obtain apoptotic cell MPs, 100 µg/ml of doxorubicin was added to the rat liver tumor cell line RH35, the rat hepatocyte cell line BRL and the rat hepatic progenitor cell line WB-F344. The apo-MPs were collected and the protein was quantified. Rats received 40 µg of MPs by spleen injection twice per week for 7 weeks. After 13 weeks, rats were sacrificed to obtain liver, heart, spleen, lung and kidney samples. Blood samples were also collected and serum was obtained for biochemical analysis. The serum levels of creatine kinase were determined. All the animal experiments were performed in accordance with the Institutional Animal Welfare Guidelines of the Second Military University, Shanghai, China.

### Histological examination

Liver samples were fixed in 4% paraformaldehyde for 48 h, then paraffin embedded, and sectioned. Hematoxylin and eosin (H&E) staining was performed. Each sample was independently assessed and scored by three pathologists.

### Assay for uptake of apo-MPs by hepatic progenitor cells in vitro and in vivo

RH35 rat liver tumor cells, BRL rat hepatocyte cells and WB-F344 rat hepatic progenitor cells were treated with doxorubicin, which emits red fluorescence. Thus, the MPs containing doxorubicin can be detected by their red fluorescence. MPs were isolated from the supernatant and then incubated with WB-F344 cells which were transfected with lentivirus expressing GFP, which shows green fluorescence. Cells were observed by fluorescence microscopy or analyzed by flow cytometry.

The isolated MPs were quantified, and 40 µg of each MP were injected into the spleen of DEN-pretreated rats. After 4 h, rats were sacrificed. Liver sample was collected. Immunofluorescence was performed using CK19 antibody (green fluorescence), then liver sections were analyzed by fluorescence microscopy to observe the uptake of red fluorescent MPs by hepatic progenitor cells in vivo.

### IHC staining and immunofluorescence

IHC analysis was performed using the following antibodies: anti-SOX9 antibody (cat.# ab185230, Abcam, Cambridge, UK), anti-rabbit secondary antibody (cat.# bs13278, Bioworld Technology, Inc., Bloomington, MN, USA), anti-CK7 antibody (cat.# ab199718, Abcam, Cambridge, UK). The detailed method has been described and published previouslyhen collected and protein was quantified [50]. Photographs of five representative fields in each section were captured to calculate the ratio of positive expression area. The density of positive staining was counted by Image-Pro Plus, version 6.2 software 9 (Media Cybernetics Inc, Bethesda, MD), as described previously in an established method [51]. Integrated optical density (IOD) of the SOX9-positive area in each image was measured, and its ratio to total area of each image was calculated as SOX9 density. For immunofluorescence, anti-caspase-3 antibody (cat.# 9664, Cell Signaling Technology Inc., Danvers, MA, U.S.A), anti-SOX9 antibody (cat.# ab185230, Abcam, Cambridge, UK), goat anti-rabbit antibody (cat.# A11011, Life Technologies, Grand Island, NY, USA ) and goat anti-rabbit antibody (cat.# A11008, Life Technologies, Grand Island, NY, USA ) were used.

### Cell viability assay

Cells were plated in 96-well plates at 5000 cells per well and allowed to grow for 24 h at 37 °C. Then various concentrations of apo-MPs were added into the cell culture medium. After 24 h incubation, cell viability was examined using a CCK-8 assay Kit (BS350B, Biosharp).

### Label-free quantitative proteomics

Label-free quantitative proteomics analysis was performed by Jingjie PTM BioLab Co Inc. (Hangzhou, China). The differentially expressed proteins between apoHPC-MPs and MPs were identified. Systematic bioinformatics analysis was then performed on all identified proteins. Analysis mainly included quantification of protein expression and differential expression analysis. Then, based on the differentially expressed proteins, protein function classification was performed including Gene Ontology (GO) enrichment and Kyoto Encyclopedia of Genes and Genomes (KEGG) enrichment.

### Statistical analysis

Statistical analysis was performed with Prism software (GraphPad Prism 8.0 software). Student’s t-test was used to compare the mean values between two groups. The data are expressed as mean ± SD. A difference of at least *p *< 0.05 was considered statistically significant. *p <0.05; **p < 0.01; ***p < 0.001; and ns, not significant.

## Supplementary Information

**Additional file 1: Table S1.** The proteins identified in apoHPC-MPs.

**Additional file 2: Figure S1.** Apoptotic WB-F344 cells release MPs. **Figure S2.** Characterization of apoHPC-MPs, apoHep-MPs and apoLTC-MPs. **Figure S3.** The observation of HPC activation in rats after treatment with or without DEN. **Figure S4.** Histological observation of tissues in rats after treatment with apoHep-MPs, apoLTC-MPs and apoHPC-MPs. **Figure S5.** ApoLTC-MPs and apoHep-MPs are internalized efficiently by RH35 cells and BRL cells, respectively. **Figure S6.** The observation of HPC activation in rats after treatment with apoHep-MPs, apoLTC-MPs and apoHPC-MPs. **Figure S7.** Doxorubicin encapsulated in apoHPCMPs has no effect on hepatocarcinogenesis. **Figure S8.** RT-apoHPC-MPs are cytotoxic to HPCs . **Figure S9.** ApoHPC-MPs are taken up by macrophages in small amounts in vivo.

## Data Availability

All data generated or analysed during this study are included in this published articles and its supplementary information files.
