# Supplementary material for: Extracellular microparticles derived from hepatic progenitor cells deliver a death signal to hepatoma-initiating cells
Source: J Nanobiotechnology. 2022 Feb 14;20:79. doi: 10.1186/s12951-022-01280-5 (PMC8842981; doi:10.1186/s12951-022-01280-5)
Supplement: Supplementary file 2 — Additional file 2: Figure S1. Apoptotic WB-F344 cells release MPs. Figure S2. Characterization of apoHPC-MPs, apoHep-MPs and apoLTC-MPs. Figure S3. The observation of HPC activation in rats after treatment with or without DEN. Figure S4. Histological observation of tissues in rats after treatment with apoHep-MPs, apoLTC-MPs and apoHPC-MPs. Figure S5. ApoLTC-MPs and apoHep-MPs are internalized efficiently by RH35 cells and BRL cells, respectively. Figure S6. The observation of HPC activation in rats after treatment with apoHep-MPs, apoLTC-MPs and apoHPC-MPs. Figure S7. Doxorubicin encapsulated in apoHPCMPs has no effect on hepatocarcinogenesis. Figure S8. RT-apoHPC-MPs are cytotoxic to HPCs . Figure S9. ApoHPC-MPs are taken up by macrophages in small amounts in vivo. [file 12951_2022_1280_MOESM2_ESM.docx]

**Supplemental Information**

**Extracellular microparticles derived from hepatic progenitor cells deliver a death signal to hepatoma-initiating cells**

Xiaojuan Hou ^1,*^, Wenting Liu^1, *^, Xue Yang^1, *^, Changchun Shao^1^, Lu Gao^1^, Li Zhang^2^, Lixin Wei^1, #^

1. Tumor Immunology and Gene Therapy Center, Third Affiliated Hospital of Second Military Medical University, Shanghai, 200438, China
2. Clinical Research Unit, Changhai Hospital, Naval Medical University, 168 Changhai Road, Shanghai 200433, China.

# Corresponding Author: Lixin Wei

# E-mail: weilixin_smmu@163.com

**Supplementary Results**

**
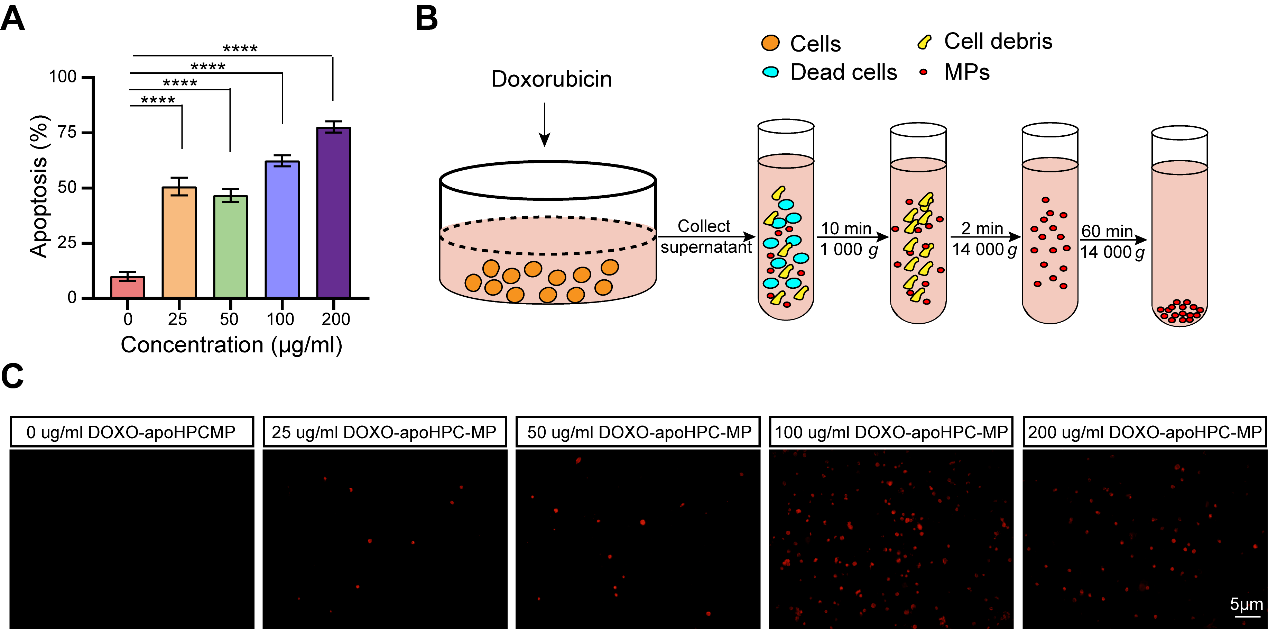
**

**Figure S1. Apoptotic WB-F344 cells release MPs**

1. WB-F344 cell were treated with 0, 25, 50, 100 or 200 μg/ml of doxorubicin for 12 h. The apoptosis of WB-F344 cells was analyzed by flow cytometry. Data are presented as mean±SD. *****p*<0.0001. (B) Experimental outline for producing MPs. (C) WB-F344 cells were treated with different doses of doxorubicin. The released apoHPC-MPs were collected. WB-F344 cells treated with 100 μg/ml of doxorubicin released large numbers of apoHPC-MPs.


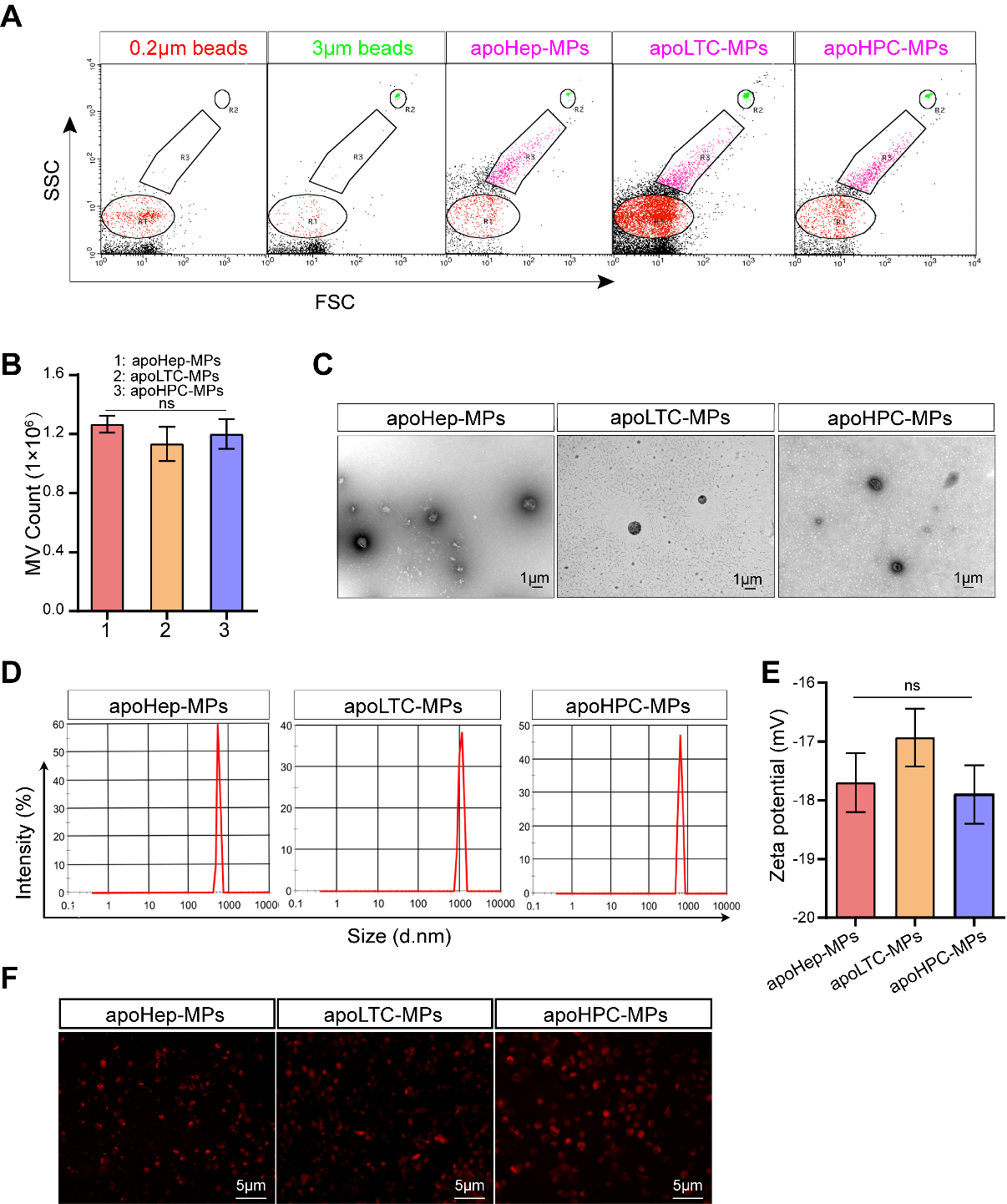


**Figure S2. Characterization of apoHPC-MPs, apoHep-MPs and apoLTC-MPs**

(A,B) WB-F344 cells, RH35 cells and BLR cells were treated with 100 μg/ml of doxorubicin for 12 h. ApoHep-MPs, apoLTC-MPs and apoHPC-MPs were isolated and counted by a flow cytometer on the basis of 0.2 μm beads and 3 μm beads. Data are presented as mean±SD. ns, not statistically significant. (C) Morphology of apoHep-MPs, apoLTC-MPs and apoHPC-MPs, as analyzed by TEM. (D) Size of apoHep-MPs, apoLTC-MPs and apoHPC-MPs, as measured by DLS. (E) Zeta potentials of apoHep-MPs, apoLTC-MPs and apoHPC-MPs, as measured by DLS. Data are presented as mean±SD. ns, not statistically significant. (F) Representative images of apoHep-MPs, apoLTC-MPs and apoHPC-MPs, as observed by fluorescence microscopy.


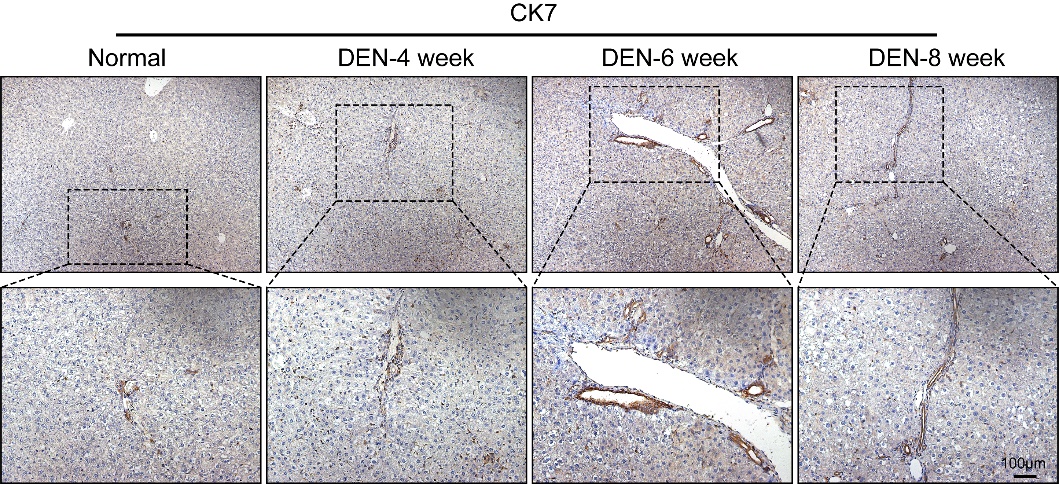


**Figure S3. The observation of HPC activation in rats after treatment with or without DEN**

The HPCs were stained with an antibody against CK7. The expression of CK7 was evaluated at different time points in DEN-induced HCC rats. Representative photographs are shown. In the normal liver (without DEN treatment), CK7 staining is faint and very scarce. The liver treated with DEN for 4 weeks shows a moderate level of CK7 staining. The liver treated with DEN for 6 weeks and 8 weeks shows a strong level of CK7 staining.


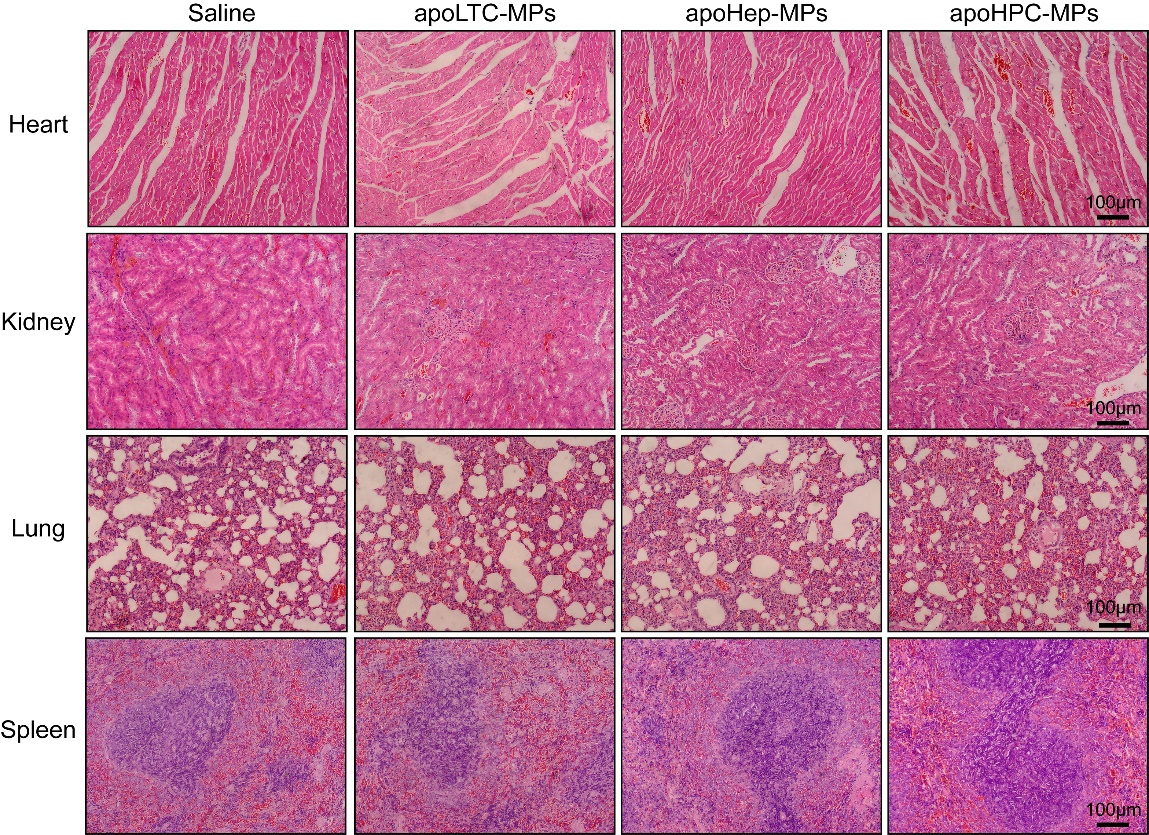


**Figure S4. Histological observation of tissues in rats after treatment with apoHep-MPs, apoLTC-MPs and apoHPC-MPs**

Sprague Dawley rats were treated orally for 6 weeks with DEN, then intrasplenically injected with 40 μg of apoHPC-MPs, apoLTC-MPs, apoHep-MPs in 200 μl saline or 200 μl of blank saline. Injections were administered twice every week for 7 weeks, in parallel with oral DEN treatment. After 13 weeks, the rats were sacrificed and sections of heart, kidney, lung and spleen tissues were stained by H&E for histological examination.


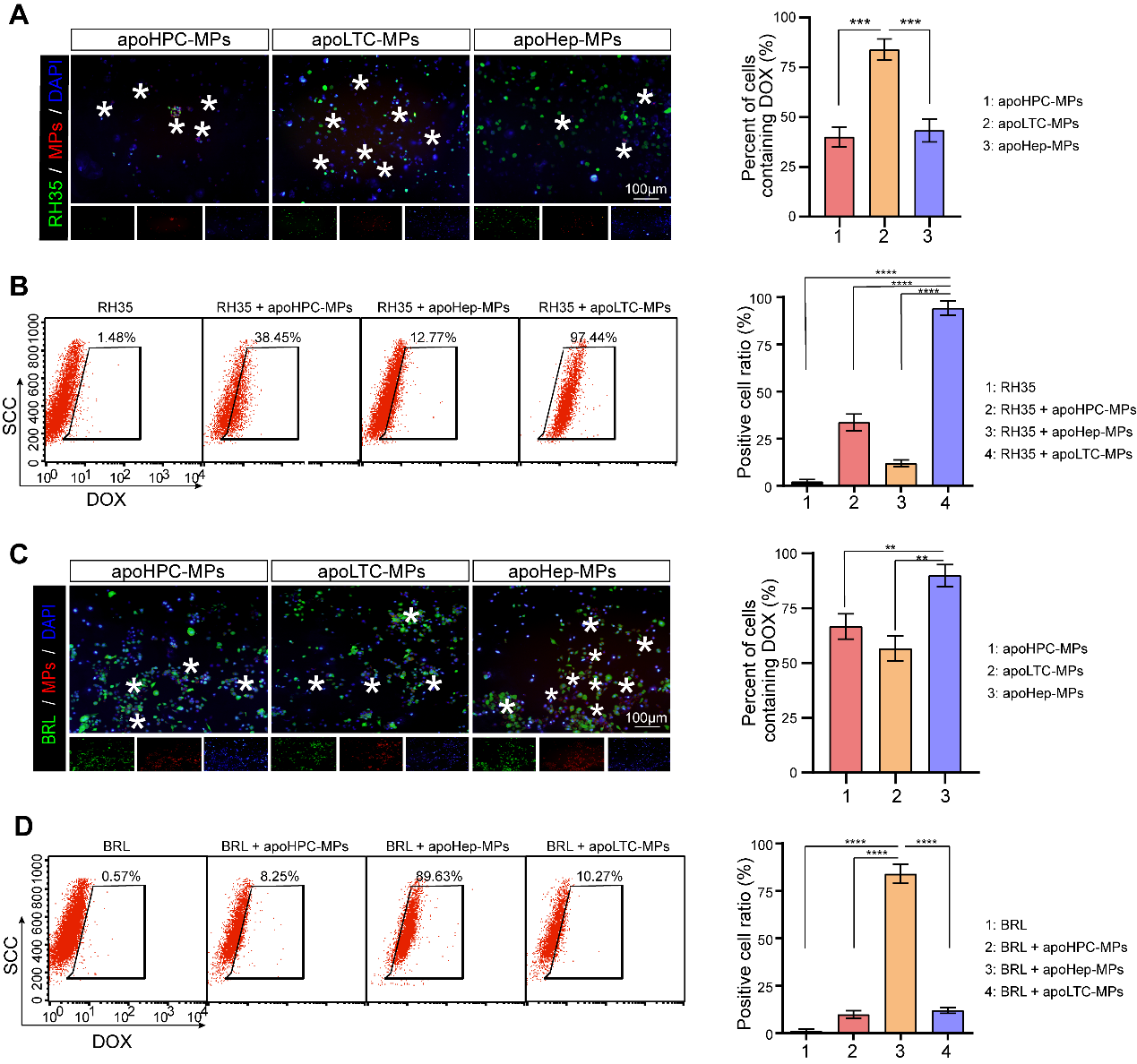


**Figure S5. ApoLTC-MPs and apoHep-MPs are internalized efficiently by RH35 cells and BRL cells, respectively**

1. RH35 cells (5×10^3^) were labelled with GFP (green fluorescence), then incubated with for 30 min with apoHPC-MPs (1×10^5^), apoLTC-MPs (1×10^5^) or apoHep-MPs (1×10^5^) containing doxorubicin (red fluorescence). The uptake of MPs by RH35 cells was observed by fluorescence microscopy. Representative images are shown in (A, left). The percentage of RH35 cells with uptake of MPs was calculated in each group. The combined data from three experiments are indicated in (A, right). Data are presented as mean±SD. ****p*<0.001. (B) ApoHPC-MPs (1×10^6^), apoLTC-MPs (1×10^6^) and apoHep-MPs (1×10^6^) containing doxorubicin (red fluorescence) were incubated with RH35 cells (3×10^5^) for 30 min. The MP-positive RH35 cells containing red fluorescence were measured by flow cytometry. Ten thousand events were collected. Representative images of flow cytometric analysis are shown in (B, left). The combined data from three experiments are shown in (B, right). Data are presented as mean±SD. *****p*<0.0001. (C) BRL cells (5×10^3^) were labeled with GFP (green fluorescence), then incubated for 30 min with apoHPC-MPs (1×10^5^), apoLTC-MPs (1×10^5^) or apoHep-MPs (1×10^5^) containing doxorubicin (red fluorescence). The uptake of MPs by BRL cells was observed by fluorescence microscopy. Representative images are shown in (C, left). The percentage of BRL cells with MP uptake (red fluorescence) was calculated in each group. The combined data from three experiments are shown in (C, right). Data are presented as mean±SD. ***p*<0.01. (D) ApoHPC-MPs (1×10^6^), apoLTC-MPs (1×10^6^) or apoHep-MPs (1×10^6^) containing doxorubicin (red fluorescence) were incubated with BRL cells (3×10^5^) for 30 min. The positive BRL cells containing red fluorescence were measured by flow cytometry. Ten thousand events were collected. Representative images of flow cytometric analysis are shown in (D, left). The combined data from three experiments are shown in (D, right). Data are presented as mean±SD. *****p*<0.0001.


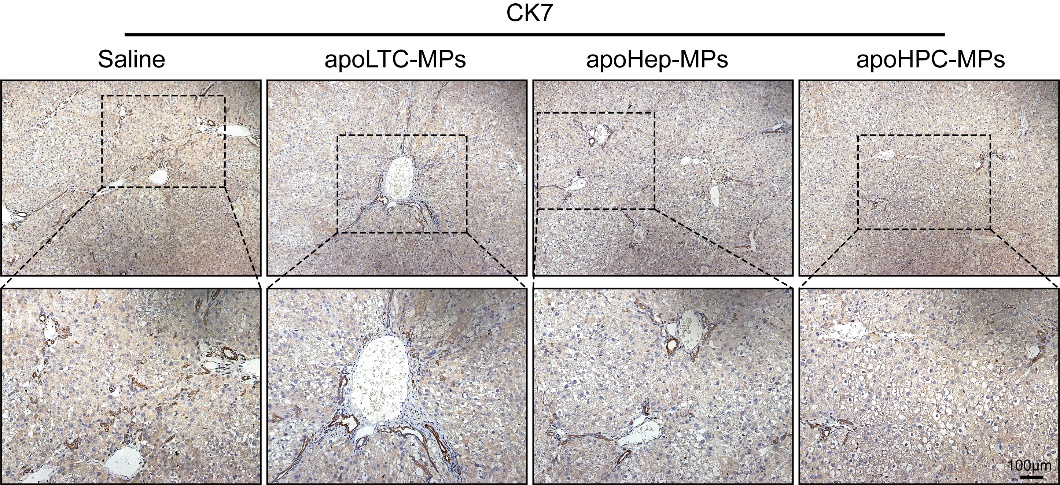


**Figure S6. The observation of HPC activation in rats after treatment with apoHep-MPs, apoLTC-MPs and apoHPC-MPs**

40 μg of apoHPC-MPs, apoHep-MPs or apoLTC-MPs were intrasplenically injected into rats, which were treated with DEN for the previous 6 weeks. Injections were administered twice every week for 4 weeks. Rats were then sacrificed, and liver tissue sections were acquired for IHC assay. The HPCs were stained with an antibody against CK7. Representative photographs are shown. In the apoHPC-MP-treated liver, CK7 staining is faint and very scarce. The apoHep-MP-treated liver shows a moderate level of CK7 staining. The other livers show strong and diffuse staining.


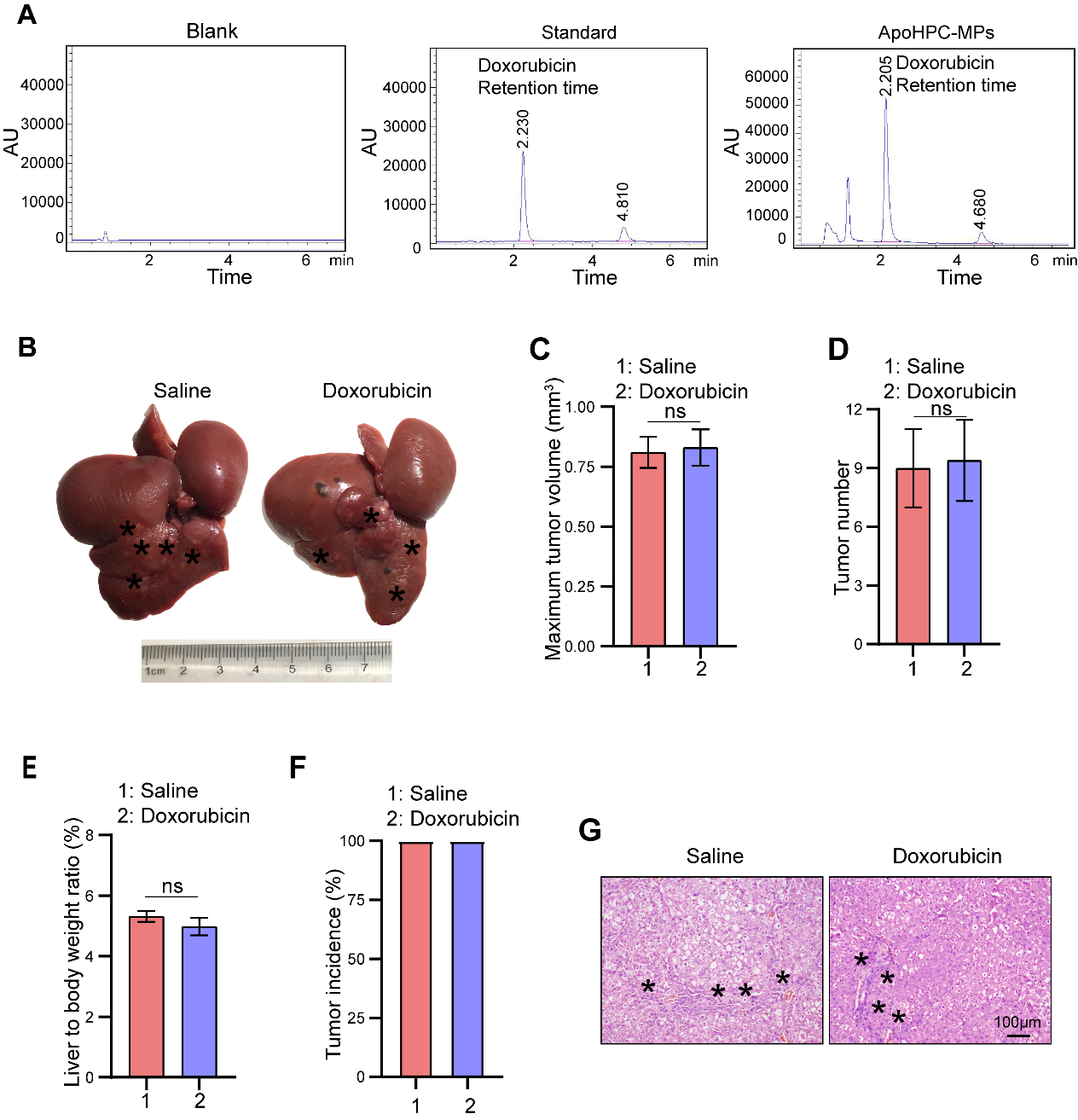


**Figure S7. Doxorubicin encapsulated in apoHPC-MPs has no effect on hepatocarcinogenesis**

1. The quantity of doxorubicin in apoHPC-MPs was analyzed by liquid chromatography-mass spectrometry (LC-MS). Doxorubicin was quantified using a Shiseido MG-C18 column (3.0×100 mm, 3.0 μm) in an Agilent 1100 HPLC system combined with mass spectrometry (Agilent 1946D). Sample concentration was calculated according to the standard curve. (B-F) Rats were treated orally with DEN for 6 weeks, then intrasplenically injected with 10 μg of free doxorubicin or saline. Injections were administered twice every week for 7 weeks. Oral DEN treatment was continued during this time. The rats were then sacrificed for tumor examination. Representative images of rat livers from the two groups are shown. Typical tumor nodes are marked by the asterisks (B). The maximum tumor volume in the two groups (C). The number of HCC nodules per liver in the two groups (D). The liver-to-body weight ratio (E). The tumor incidence in the two groups (F). Data are presented as mean±SD. ns, not statistically significant. (G) H&E-stained liver sections showing the histological structure and inflammatory response in the indicated groups. Black asterisks indicate accumulation of inflammatory cells.


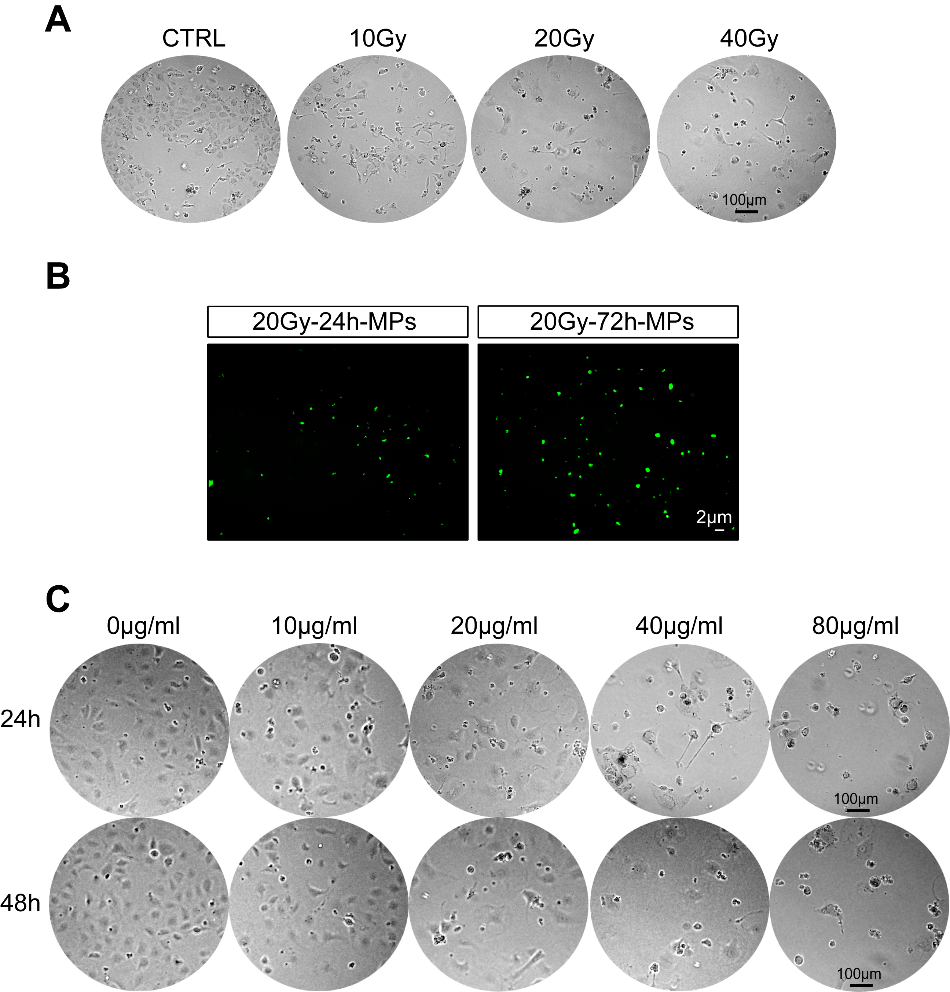


**Figure S8. RT-apoHPC-MPs are cytotoxic to HPCs**

1. WB-F344 cells were treated with various doses of radiation. Cell killing was observed by microscopy. Representative images from three independent experiments are shown here. (B) WB-F344 cells were treated with 20 Gy of radiation, then cultured for another 24 h or 72 h. The MPs were then isolated, labeled with the green fluorescent dye PKH67 (PKH67 Green Fluorescent Cell Linker Mini Kit for General Cell Membrane Labeling), and observed by fluorescence microscopy. The images shown here are representative of three independent experiments. (C) WB-F344 cells were treated with various concentrations of RT-apoHPC-MPs for 24 h or 48 h. Cell killing was observed by microscopy. The images shown here are representative of three independent experiments.


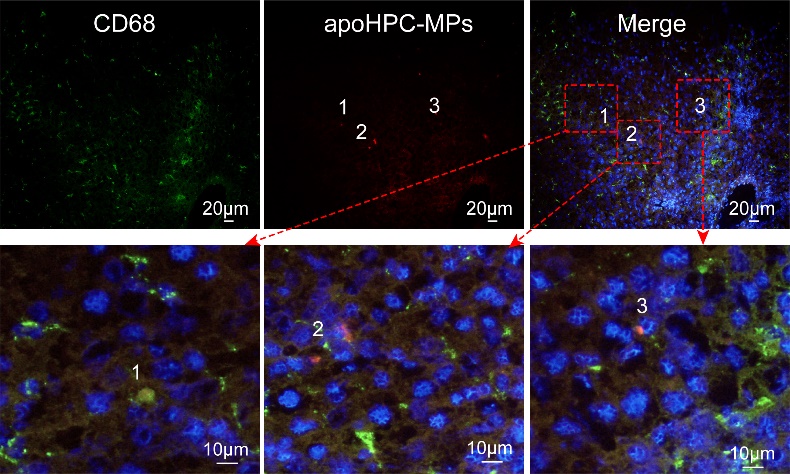


**Figure S9. ApoHPC-MPs are taken up by macrophages in small amounts *in vivo*** ApoHPC-MPs (1×10^7^) were intrasplenically injected into DEN-treated rats, and liver slices were acquired for fluorescence detection 30 minutes after injection. Macrophages were recognized by antibodies against CD68 (green). Cell nuclei were stained with DAPI. The images shown here are representative of three independent experiments.
